# Gestational age and device-measured physical activity in adolescents and adults: A linkage of the HUNT Study and Medical Birth Registry

**DOI:** 10.1371/journal.pone.0357277

**Published:** 2026-09-23

**Authors:** Kristina Anna Djupvik Aakvik, Silje Dahl Benum, Marjaana Tikanmäki, Stian Lydersen, Atle Kongsvold, Paul Jarle Mork, Eero Kajantie, Kari Anne I. Evensen

**Affiliations:** 1 Department of Clinical and Molecular Medicine, Norwegian University of Science and Technology, Trondheim, Norway; 2 St. Olavs hospital, Trondheim University Hospital, Trondheim, Norway; 3 Clinical Medicine Research Unit Oulu, Oulu University Hospital and University of Oulu, Oulu, Finland; 4 Public Health Promotion Unit, Finnish Institute for Health and Welfare, Helsinki, Finland; 5 Department of Mental Health, Norwegian University of Science and Technology, Trondheim, Norway; 6 Department of Public Health and Nursing, Norwegian University of Science and Technology, Trondheim, Norway; 7 Children’s Hospital, Helsinki University Hospital and University of Helsinki, Helsinki, Finland; 8 Department of Rehabilitation Science and Health Technology, Oslo Metropolitan University, Oslo, Norway; 9 Children’s Clinic, St. Olavs hospital, Trondheim University Hospital, Trondheim, Norway; UBC: The University of British Columbia, CANADA

## Abstract

There is limited knowledge of the relationship between gestational age and physical activity assessed by accelerometers in population-based cohort studies. We examined how gestational age predicts physical activity among adolescents and adults. Data from 12 896 participants (13–52 years) with physical activity assessed by two tri-axial accelerometers for seven days in the population-based Trøndelag Health Study (HUNT Study) were linked to the Medical Birth Registry of Norway. Metabolic equivalent of task (MET) min/day and min/day in total, moderate to vigorous, and light physical activity were calculated. Effects of gestational age (in weeks) and birth weight (in grams) on physical activity were modelled using fractional polynomials and linear regression. Gestational age predicted MET min/day in light physical activity (B = 1.53, 95% CI: 0.02 to 3.07) in the total sample, especially among females (B = 2.14, 95% CI: 0.33 to 3.93), and in total physical activity among adolescents (B = 2.44, 95% CI: 0.16 to 4.88). Gestational age predicted min/day in total physical activity in the total sample (B = 0.99, 95% CI: 0.07 to 1.90), among females (B = 1.25, 95% CI: 0.11 to 2.40) and adolescents (B = 1.06, 95% CI: 0.02 to 2.12). There were no non-linear or linear effects of birth weight on physical activity. Shorter gestation predicted less physical activity in the total sample, among females, and in adolescents. The associations with total physical activity are by and large consistent with previous observations that individuals born preterm with very low birth weight have lower rates of physical activity.

## Introduction

About 10% of all live births worldwide occur preterm (<37 weeks of gestation) [1,2]. Preterm birth is associated with medical disabilities [3], as well as cardiometabolic [4], respiratory [5], mental health [6] and psychiatric disorders [7] compared with term birth (≥37 weeks of gestation). These outcomes may manifest during childhood, adolescence or adulthood.

Physical activity (PA) is beneficial for several health outcomes [8]. Given the high risk of adverse health in preterm populations [3–7], PA is particularly important for individuals born preterm. More than 8 out of 10 individuals born preterm are at the higher end of the gestational age range (32 to <37 weeks of gestation) [1], indicating that PA could have significant impact on a population-level. A meta-analysis including 13 Nordic cohorts found that individuals with low and high birth weight engaged in less PA during adolescence and adulthood compared to individuals with normal birth weight, as measured by self-report [9]. Additionally, an individual participant data (IPD) meta-analysis found that individuals born very preterm or with very low birth weight (VLBW) reported less time in moderate to vigorous PA (MVPA) compared with controls, especially among women [10].

Although PA has been examined by self-report in adolescents and adults born preterm [10–16], assessments with accelerometers remains limited [16–19], and no studies have explored PA across gestational age in this population. The few published cohort studies which have measured PA using accelerometers in adults born preterm are based on relatively small cohorts and most of them only include the lower end of gestation. In a recent study, MVPA differed between adults born preterm with VLBW and a term-born control group in the Helsinki Study of Very Low Birth Weight Adults (HeSVA) and Norwegian University of Science and Technology Low Birth Weight in a Lifetime Perspective study (NTNU LBW Life) cohorts [19]. In contrast, MVPA did not differ between adults born with VLBW in the HeSVA cohort compared with controls [17], nor did PA among participants born early preterm (<34 weeks) or late preterm (34–36 weeks) compared with term-born controls in the ESTER Preterm Birth Study (ESTER) cohort [18].

Based on previous findings, there is limited knowledge of PA assessed by accelerometry in large population-based cohorts of adolescents and adults born preterm. No studies have investigated the shape of the association between a wide range of gestational ages and PA assessed by accelerometry. The aim of this study was therefore to examine how gestational age predicts PA in a large population-based cohort of adolescents and adults.

## Methods

### Study design

In this population-based register linkage study, we included individuals who participated in the fourth wave of the Trøndelag Health Study (HUNT Study), Norway, in 2017–2019. The data collection comprised questionnaires, interviews, clinical tests, laboratory measurements, biological samples and accelerometer data [20]. The HUNT4 Survey has been described in more detail previously [20,21]. Data from the Young-HUNT4 Survey (13–19 years) and HUNT4 Survey (≥20 years) were linked to the Medical Birth Registry of Norway (MBRN), which includes information of all live births in Norway from year 1967 [22]. The linkage was performed by encrypted unique identification codes. All data were accessed for research purposes on 08/07/2022.

### Study population

**Fig 1:**
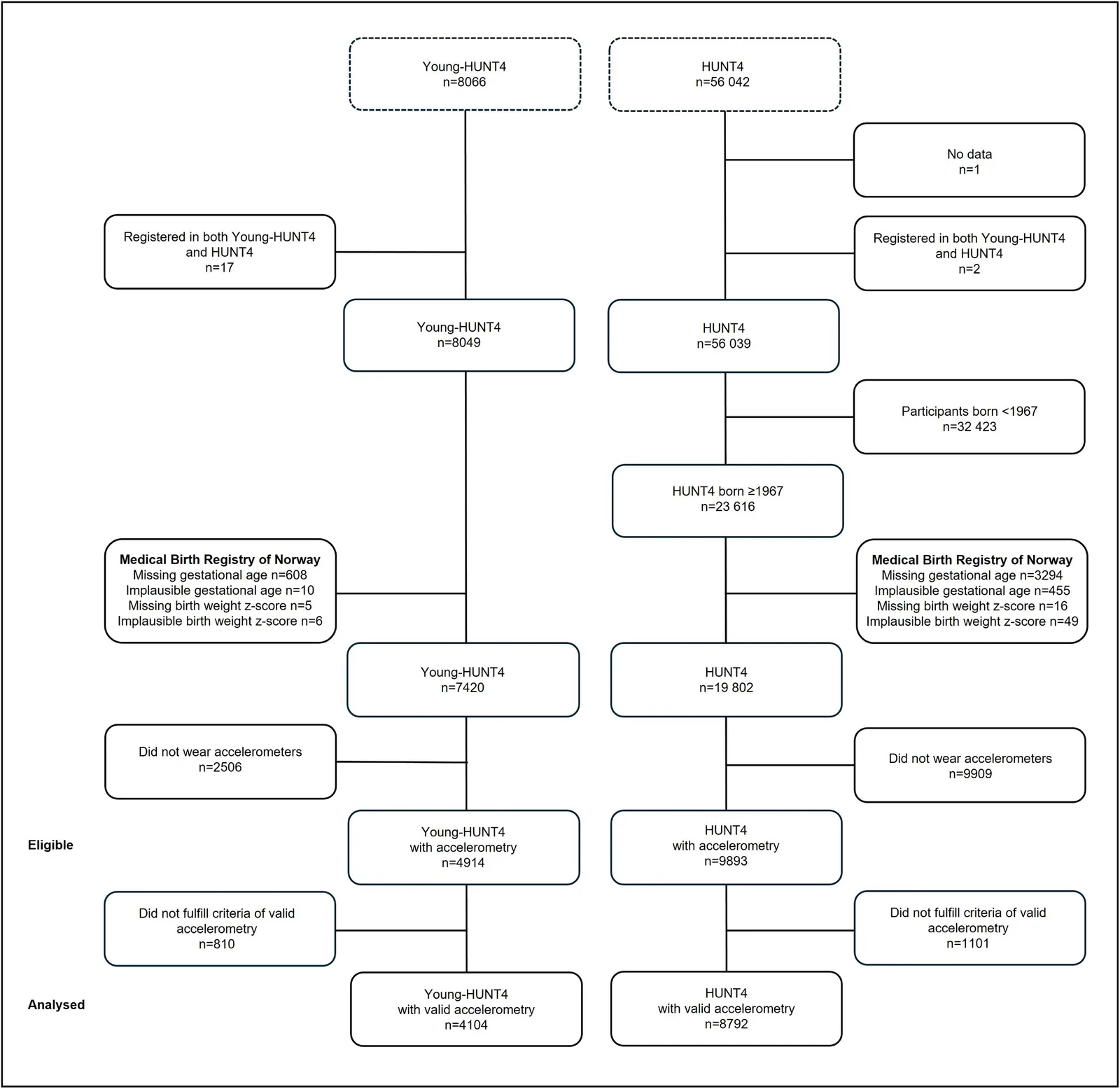
Flow of the study participants. Medical Birth Registry of Norway (MBRN): Participants born ≥1967, corresponding to the establishment of the MBRN. Implausible birth weight z-score: <−6 or >4. Implausible gestational age: <22.0 weeks or >43.6 weeks. Inclusion criteria for accelerometer data: Valid days: >23 hours of recording and >5 min in any dynamic activity (running, cycling, brisk walking, moderate walking, slow walking). Valid data: ≥2 valid weekdays and ≥1 valid weekend day(s). Abbreviations: HUNT4 = Trøndelag Health Study 2017–2019, Survey 4; Young-HUNT4 = Trøndelag Health Study 2017–2019 (13–19 years), Survey 4.

Flow of the 8066 adolescents from the Young-HUNT4 Survey, and 56 042 adults from the HUNT4 Survey, are shown in Fig 1. The participants were included if they had a valid date of participation for the given survey, were born ≥1967, corresponding to the establishment of the MBRN, and had valid accelerometry data, including valid recording for at least two weekdays and one weekend day. A valid recording day was defined as >23 hours of recording and >5 min in any dynamic behavior or combined (running, cycling, brisk walking, moderate walking or slow walking) were required. Exclusion criteria were missing gestational age, implausible gestational age (<22.0 or >43.6 weeks), missing birth weight z-score based on growth curves from Skjaerven [23], implausible birth weight z-score (<−6 or >4), no accelerometry, or not fulfilling the criteria of valid accelerometry data. We did not impute missing data.

Of the 8066 adolescents in the Young-HUNT4 Survey, 17 were excluded as they were registered in both Young-HUNT4 and HUNT4. When linked to MBRN, 608 were excluded due to missing gestational age, 10 had implausible gestational age (<22.0 or >43.6 weeks), five had missing birth weight z-score and six had implausible birth weight z-score (<−6 or >4), leaving 7420 participants with valid data from MBRN. Of those, 2506 did not wear accelerometers, leaving 4914 participants with accelerometry. Furthermore, 810 participants did not fulfill the criteria of valid accelerometry, resulting in 4104 participants with valid data.

Of the 56 042 adults in the HUNT4 Survey, one had no data and two were excluded as they were registered in both Young-HUNT4 and HUNT4. A total of 32 423 participants were excluded as they were born <1967, leaving 23 616 participants who were born ≥1967. Out of these, 3294 had missing gestational age, 455 had implausible gestational age (<22.0 or >43.6 weeks), 16 had missing birth weight z-score and 49 had implausible birth weight z-score (<−6 or >4), leaving 19 802 participants. Furthermore, 9909 did not wear accelerometers, leaving 9893 participants with accelerometry. Of those, 1101 participants did not fulfill the criteria of valid accelerometry, resulting in 8792 participants with valid data.

### Exposure variables

Days of gestational age at birth were estimated from ultrasonography, last menstrual period or by day of embryo transfer by MBRN and converted into weeks and days. Birth weight was reported in grams. Table 1 shows descriptive statistics by groups of completed weeks of gestational age at birth: < 32 weeks (very preterm), 32–33 weeks (moderately preterm), 34–36 weeks (late preterm), 37–38 weeks (early term), 39–40 weeks (full term), 41 weeks (late term) and 42–43.6 weeks (post term) [24].

**Table 1:** Background characteristics of adolescents and adults by gestational age group.

Total sample (n = 12 896)	Preterm (n = 621)						Term-born (n = 12 275)							
	Very preterm (<32 weeks) n = 73		Moderately preterm (32–33 weeks) n = 92		Late preterm (34–36 weeks) n = 456		Early term (37–38 weeks) n = 1798		Full term (39–40 weeks) n = 6120		Late term (41 weeks) n = 2745		Post term (42–43.6 weeks) n = 1612	
Gestational age (weeks), mean (SD)	29.5	(1.8)	33.0	(0.5)	35.8	(0.8)	38.2	(0.5)	40.0	(0.6)	41.4	(0.3)	42.5	(0.4)
Birth weight z-score, mean (SD)	−0.3	(1.1)	−0.2	(1.4)	−0.1	(1.2)	−0.1	(1.1)	0.02	(1.0)	0.04	(1.0)	0.02	(1.0)
Birth weight (g), mean (SD)	1326	(428)	2077	(524)	2762	(563)	3269	(496)	3592	(464)	3737	(479)	3760	(476)
Female, n (%)	44	(60.3)	53	(57.6)	260	(57.0)	1063	(59.1)	3725	(60.9)	1640	(59.7)	967	(60.0)
Birthyear in categories, n (%)														
1967-1976	15	(20.5)	23	(25.0)	101	(22.1)	458	(25.5)	1871	(30.6)	883	(32.2)	546	(33.9)
1977-1986	7	(9.6)	7	(7.6)	78	(17.1)	266	(14.8)	1207	(19.7)	594	(21.6)	409	(25.4)
1987-1996	11	(15.1)	12	(13.0)	81	(17.8)	238	(13.2)	925	(15.1)	466	(17.0)	287	(17.8)
1997-2005	40	(54.8)	50	(54.3)	196	(43.0)	836	(46.5)	2117	(34.6)	802	(29.2)	370	(23.0)
**Young-HUNT4** (n = 4104)	Preterm (n = 267)						Term-born (n = 3837)							
	Very preterm (<32 weeks) n = 36		Moderately preterm (32–33 weeks) n = 47		Late preterm (34–36 weeks) n = 184		Early term (37–38 weeks) n = 797		Full term (39–40 weeks) n = 1976		Late term (41 weeks) n = 737		Post term (42–43.6 weeks) n = 327	
Gestational age (weeks), mean (SD)	29.0	(1.9)	33.0	(0.4)	35.8	(0.9)	38.2	(0.5)	40.0	(0.6)	41.4	(0.3)	42.3	(0.3)
Birth weight z-score, mean (SD)	−0.4	(1.0)	−0.5	(1.1)	−0.3	(1.0)	0.04	(1.0)	0.2	(1.0)	0.3	(1.0)	0.4	(0.9)
Birth weight (g), mean (SD)	1216	(426)	1950	(421)	2675	(485)	3319	(480)	3671	(441)	3870	(468)	3973	(445)
Female, n (%)	25	(69.4)	24	(51.1)	106	(57.6)	487	(61.1)	1259	(63.7)	405	(55.0)	163	(49.8)
Age at assessment in years, mean (SD)	15.4	(1.6)	15.2	(2.0)	16.2	(1.7)	15.8	(1.8)	15.9	(1.8)	16.0	(1.8)	16.0	(1.9)
Height in cm^a^, mean (SD)	163.5	(8.8)	167.0	(8.9)	168.9	(9.5)	168.3	(8.9)	169.0	(8.8)	170.2	(9.1)	170.4	(9.0)
Weight in kg^b^, mean (SD)	57.5	(12.0)	59.6	(11.3)	65.6	(16.9)	62.2	(13.8)	63.2	(13.9)	63.9	(14.8)	64.9	(13.8)
Body mass index^c^, mean (SD)	21.4	(3.6)	21.3	(3.0)	22.9	(4.9)	21.8	(4.0)	22.0	(4.0)	22.0	(4.2)	22.3	(3.9)
**HUNT4** (n = 8792)	Preterm (n = 354)						Term-born (n = 8438)							
	Very preterm (<32 weeks) n = 37		Moderately preterm (32–33 weeks) n = 45		Late preterm (34–36 weeks) n = 272		Early term (37–38 weeks) n = 1001		Full term (39–40 weeks) n = 4144		Late term (41 weeks) n = 2008		Post term (42–43.6 weeks) n = 1285	
Gestational age (weeks), mean (SD)	30.0	(1.5)	33.0	(0.6)	35.8	(0.8)	38.2	(0.5)	40.1	(0.5)	41.4	(0.3)	42.5	(0.4)
Birth weight z-score, mean (SD)	−0.3	(1.1)	0.2	(1.5)	0.02	(1.3)	−0.2	(1.1)	−0.07	(1.0)	−0.06	(1.0)	−0.09	(1.0)
Birth weight (g), mean (SD)	1433	(406)	2209	(589)	2822	(603)	3229	(505)	3554	(470)	3688	(474)	3706	(469)
Female, n (%)	19	(51.4)	29	(64.4)	154	(56.6)	576	(57.5)	2466	(59.5)	1235	(61.5)	804	(62.6)
Age at assessment in years, mean (SD)	35.0	(10.0)	37.3	(10.3)	37.2	(9.3)	38.1	(9.4)	38.3	(9.1)	38.0	(9.0)	38.2	(8.9)
Height in cm^d^, mean (SD)	171.9	(10.5)	170.7	(9.6)	172.4	(8.8)	172.9	(9.0)	172.9	(9.1)	172.6	(8.8)	172.4	(8.9)
Weight in kg (InBody)^e^, mean (SD)	74.5	(18.3)	79.5	(16.7)	78.9	(16.3)	79.0	(16.0)	79.3	(16.6)	79.0	(16.4)	79.4	(16.7)
Body mass index^f^, mean (SD)	24.9	(4.4)	27.2	(5.3)	26.5	(4.8)	26.4	(4.5)	26.5	(4.7)	26.5	(4.7)	26.7	(4.8)

Abbreviations: HUNT4 = Trøndelag Health Study 2017–2019, Survey 4; SD = standard deviation; Young-HUNT4 = Trøndelag Health Study 2017–2019 (13–19 years), Survey 4.

^a^Data missing for one moderately preterm participant, one late preterm participant, five early term participants, twelve full term participants, eight late term participants and two post term participants.

^b^Data missing for one moderately preterm participant, one late preterm participant, four early term participants, twelve full term participants, seven late term participants and two post term participants.

^c^Data missing for one moderately preterm participant, one late preterm participant, five early term participants, twelve full term participants, eight late term participants and two post term participants.

^d^Data missing for one late preterm participant, four full term participants, three late term participants and three post term participants.

^e^Data missing for six full term participants, three late term participants and three post term participants.

^f^Data missing for one late preterm participant, six full term participants, four late term participants and three post term participants.

### Outcome variables

To assess PA, the participants were asked to wear two tri-axial accelerometers (AX3, Axivity. Newcastle, United Kingdom) attached to the skin on the right thigh and the lower back for seven days. The data collection procedure has been described in detail elsewhere [25,26]. In brief, the OmGui software (version 1.0.0.37; Open Movement, Newcastle University, United Kingdom) was used to configure the accelerometers (recorded 50 Hz with a bandwidth of 8G). Data from both accelerometers was synchronized into a CSV file (5 second window segmentation) and fed into the eXtreme Gradient Boosting (XGBoost) machine learning model. The model has previously been validated with a 96% accuracy [27–29]. For the given study, the following PA behaviors were predicted; running, cycling, brisk walking, moderate walking, slow walking and standing [28,29].

Total time spent in PA included min/day in running, cycling, brisk walking, moderate walking, slow walking and standing. The total PA was categorized into MVPA (running, cycling, brisk walking, moderate walking) and light PA (slow walking, standing). We calculated metabolic equivalent of task (MET) min/day of the PA behaviors by multiplying min/day for each behavior by conversion factors for MET, running = 6.0, cycling = 4.0, brisk walking = 4.5, moderate walking = 3.5, slow walking = 2.8 and standing = 1.5 (S1 Table) [30].

### Background variables

Background characteristics include sex, birth year in categories, age, height and weight at assessment, and body mass index (BMI). Height was measured in cm, and weight was measured in kg by a bioelectrical impedance machine (InBody 770, Cerritos, CA, USA). BMI was calculated (kg/m^2^).

### Ethical considerations

The linkage between the HUNT Study and MBRN was approved by the Regional Committee for Medical and Health Research Ethics (REK) in Central Norway (ref. no. 429458), based on the broad written informed consent obtained in the HUNT Study (REK in Central Norway; ref. nr. 67445). The project has established a lawful basis for the processing of data from the MBRN in accordance with the General Data Protection Regulation (GDPR), Articles 6(1)(a) and 9(2)(j).

In addition, the Norwegian Institute of Public Health assessed the application and confirmed that the project falls within the purpose of the MBRN and that the conditions for data access were fulfilled, in accordance with Sections 19(a–e) of the Health Register Act and Sections 1–3 of the Regulations relating to the MBRN. All data were pseudonymized before researcher access.

### Statistical analyses

Main outcome variables were MET min/day and min/day in total PA, MVPA and light PA. Potential non-linear effects of gestational age (in weeks), with adjustment for age and sex, were modelled using fractional polynomials of order two, which could be more appropriate to address the relationship between variables due to its flexibility [31] and inclusion of several dimensions. In case of no effects by the non-linear model, we performed linear regression with MET min/day and min/day in the PA categories as outcome, gestational age (in weeks) as continuous independent variable, adjusted for age and sex. Plurality (singleton vs. multiple birth) was not accounted for, as their relatively low prevalence suggests that any impact on the results is likely to be limited. Due to some deviations from normality, we used bootstrapping with B = 2000 bootstrap samples and the bias-corrected and accelerated method. A significance level of 0.05 was used. The statistical software Stata MP 17 (64-bit) Windows was used to perform fractional polynomials and IBM SPSS Statistics (version 28.0.0) Windows was used for the linear regression analyses. Subgroup analyses were performed for adolescents and adults, and for females and males, as PA may differ by sex, both within the total sample and among adolescents and adults separately.

### Supplementary analyses

Non-participants were categorized as those who took part in the Young-HUNT4 Survey or the HUNT4 Survey with valid date of participation for the given survey, were born ≥1967, corresponding to the establishment of the MBRN, had valid gestational age and birth weight z-score, but who had no accelerometry data. Background characteristics of both participants and non-participants are presented for the total sample in S2-S3 Table.

For adolescents, information about motor impairment, visual impairment, hearing impairment and leisure-time exercise was obtained by self-report. Adults were asked whether they have any longstanding illness or injury, and leisure-time exercise was assessed by questions related to frequency, intensity and duration. Background characteristics of participants and non-participants are presented for adolescents in S4-S5 Table and for adults in S6-S7 Table.

Supplementary analyses with potential non-linear effects of birth weight (in grams) on MET min/day and min/day in total PA, MVPA and light PA as outcome, adjusted for age and sex, were also performed in the total sample and in the subsamples. Linear regression analyses with MET min/day and min/day in the PA categories as outcome and birth weight (in 100 grams) as continuous independent variable, with adjustments for age and sex, were also performed. Supplementary linear regression analyses with MET min/day and min/day in the PA categories as outcome, gestational age in weeks as continuous independent variable, and additional adjustment for BMI were performed to evaluate the robustness of the findings [32].

## Results

### Background characteristics

Background characteristics of the study participants by gestational age group in the total sample as well as for Young-HUNT4 and HUNT4 are shown in Table 1. Accelerometry data of the study participants by gestational age group are shown in Table 2.

**Table 2:** Accelerometry data of adolescents and adults by gestational age group.

Total sample (n = 12 896)	Preterm (n = 621)						Term-born (n = 12 275)							
	Very preterm (<32 weeks) n = 73		Moderately preterm (32–33 weeks) n = 92		Late preterm (34–36 weeks) n = 456		Early term (37–38 weeks) n = 1798		Full term (39–40 weeks) n = 6120		Late term (41 weeks) n = 2745		Post term (42–43.6 weeks) n = 1612	
	**Mean**	**(SD)**	**Mean**	**(SD)**	**Mean**	**(SD)**	**Mean**	**(SD)**	**Mean**	**(SD)**	**Mean**	**(SD)**	**Mean**	**(SD)**
MET min/day in total PA	720.1	(188.5)	765.6	(183.9)	737.0	(202.9)	750.0	(197.5)	765.4	(203.1)	768.6	(197.3)	782.6	(199.1)
MET min/day in MVPA	171.8	(76.3)	180.0	(94.2)	159.3	(87.8)	166.8	(89.1)	163.0	(92.8)	159.7	(88.8)	157.7	(93.3)
MET min/day in light PA	548.3	(159.3)	585.6	(159.3)	577.7	(175.6)	583.2	(170.4)	602.4	(173.7)	608.8	(171.7)	624.9	(171.9)
Min/day in total PA	354.2	(95.5)	373.0	(92.7)	366.0	(103.2)	370.5	(100.0)	381.0	(102.2)	384.4	(101.3)	392.8	(101.4)
Min/day in MVPA	41.7	(17.2)	43.3	(21.4)	38.6	(20.1)	40.3	(20.4)	39.5	(21.3)	38.8	(20.4)	38.4	(21.3)
Min/day in light PA	312.5	(90.1)	329.7	(90.5)	327.3	(99.7)	330.2	(96.4)	341.5	(98.3)	345.6	(98.2)	354.5	(98.0)
**Young-HUNT4** (n = 4104)	Preterm (n = 267)						Term-born (n = 3837)							
	Very preterm (<32 weeks) n = 36		Moderately preterm (32–33 weeks) n = 47		Late preterm (34–36 weeks) n = 184		Early term (37–38 weeks) n = 797		Full term (39–40 weeks) n = 1976		Late term (41 weeks) n = 737		Post term (42–43.6 weeks) n = 327	
	**Mean**	**(SD)**	**Mean**	**(SD)**	**Mean**	**(SD)**	**Mean**	**(SD)**	**Mean**	**(SD)**	**Mean**	**(SD)**	**Mean**	**(SD)**
MET min/day in total PA	658.9	(179.2)	714.0	(155.1)	642.9	(176.8)	679.7	(174.6)	677.0	(172.3)	675.8	(169.6)	681.0	(164.8)
MET min/day in MVPA	186.4	(76.8)	212.7	(100.7)	179.6	(91.6)	191.0	(92.8)	191.1	(93.5)	192.7	(94.7)	194.3	(91.4)
MET min/day in light PA	472.4	(132.7)	501.3	(94.0)	463.4	(113.6)	488.7	(119.7)	485.9	(118.5)	483.1	(114.2)	486.7	(114.4)
Min/day in total PA	315.2	(85.0)	332.0	(63.6)	305.1	(74.4)	322.0	(78.2)	320.9	(76.2)	319.5	(74.9)	321.3	(72.6)
Min/day in MVPA	44.4	(17.4)	49.9	(22.4)	42.9	(20.8)	45.3	(21.0)	45.3	(20.9)	45.8	(21.3)	45.9	(20.2)
Min/day in light PA	270.8	(76.1)	282.1	(55.5)	262.2	(63.3)	276.7	(69.2)	275.6	(67.8)	273.8	(66.4)	275.4	(65.0)
**HUNT4** (n = 8792)	Preterm (n = 354)						Term-born (n = 8438)							
	Very preterm (<32 weeks) n = 37		Moderately preterm (32–33 weeks) n = 45		Late preterm (34–36 weeks) n = 272		Early term (37–38 weeks) n = 1001		Full term (39–40 weeks) n = 4144		Late term (41 weeks) n = 2008		Post term (42–43.6 weeks) n = 1285	
	**Mean**	**(SD)**	**Mean**	**(SD)**	**Mean**	**(SD)**	**Mean**	**(SD)**	**Mean**	**(SD)**	**Mean**	**(SD)**	**Mean**	**(SD)**
MET min/day in total PA	779.7	(180.1)	819.4	(197.4)	800.7	(194.7)	805.9	(197.0)	807.6	(203.1)	802.6	(195.9)	808.4	(198.9)
MET min/day in MVPA	157.6	(73.9)	145.8	(73.4)	145.6	(82.5)	147.5	(81.0)	149.7	(89.5)	147.6	(83.4)	148.4	(91.4)
MET min/day in light PA	622.1	(149.3)	673.6	(166.6)	655.1	(167.8)	658.4	(167.3)	657.9	(168.3)	655.0	(166.1)	660.1	(166.3)
Min/day in total PA	392.1	(90.7)	415.8	(99.3)	407.1	(99.7)	409.1	(98.4)	409.7	(100.6)	408.3	(99.2)	411.0	(99.6)
Min/day in MVPA	39.0	(16.9)	36.4	(17.9)	35.7	(19.2)	36.3	(19.0)	36.8	(20.9)	36.3	(19.5)	36.4	(21.1)
Min/day in light PA	353.2	(84.5)	379.4	(93.7)	371.4	(95.8)	372.8	(93.8)	372.9	(95.0)	372.0	(94.7)	374.6	(94.8)

Abbreviations: HUNT4 = Trøndelag Health Study 2017–2019, Survey 4; MET = Metabolic equivalent of task; MVPA = moderate to vigorous physical activity; PA = physical activity; SD = standard deviation; Young-HUNT4 = Trøndelag Health Study 2017–2019 (13–19 years), Survey 4.

### Non-linear effects

**Fig 2:**
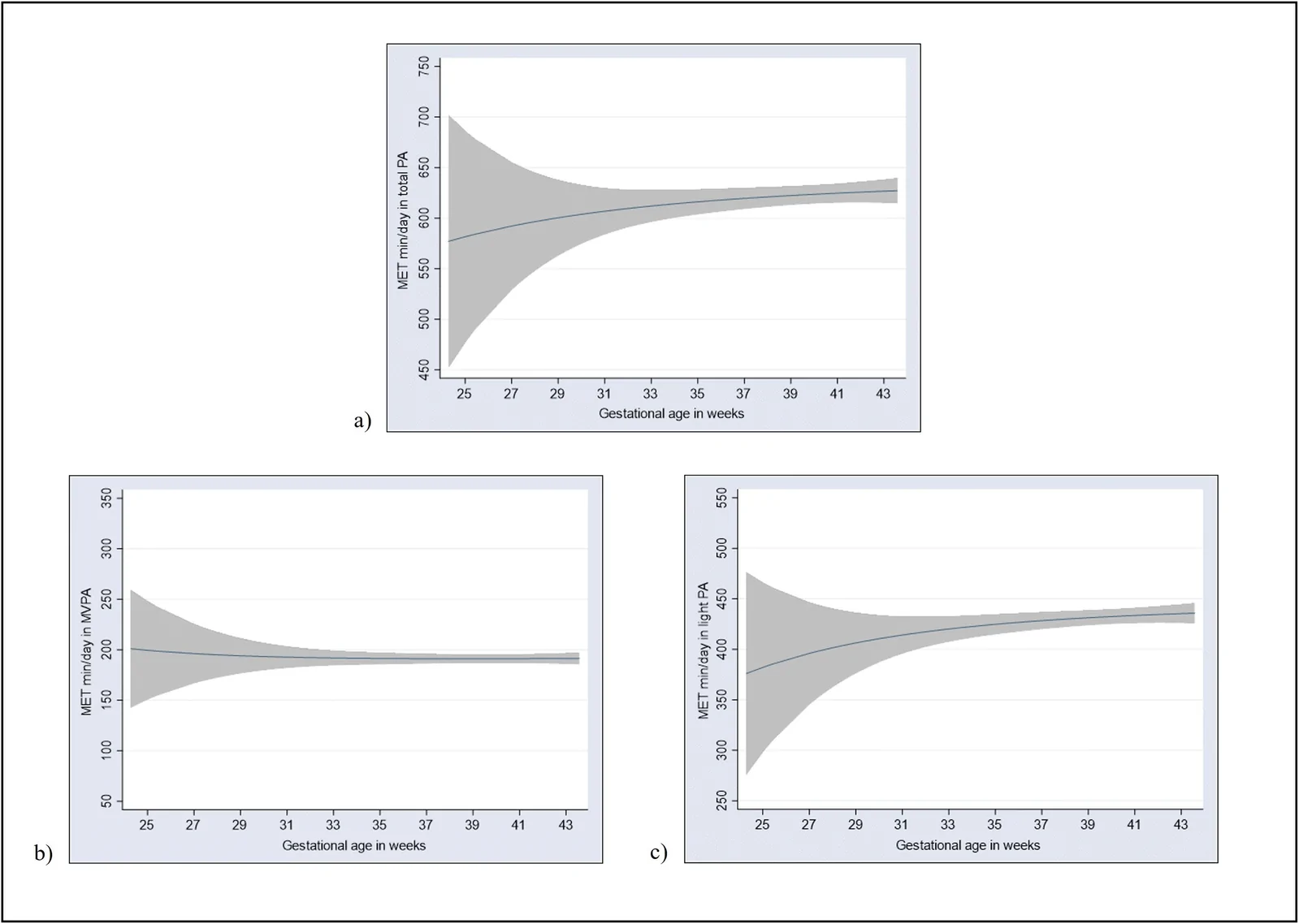
MET min/day in PA categories by gestational age among adolescents and adults. The marked line shows MET min/day for **a)** total PA, **b)** MVPA and **c)** light PA from linear regression with fractional polynomials including 95% CI (shaded area), adjusted for age and sex (n = 12 896). Abbreviations: CI=Confidence interval; MET = metabolic equivalent of task; MVPA = moderate to vigorous physical activity; PA = physical activity.

**Fig 3:**
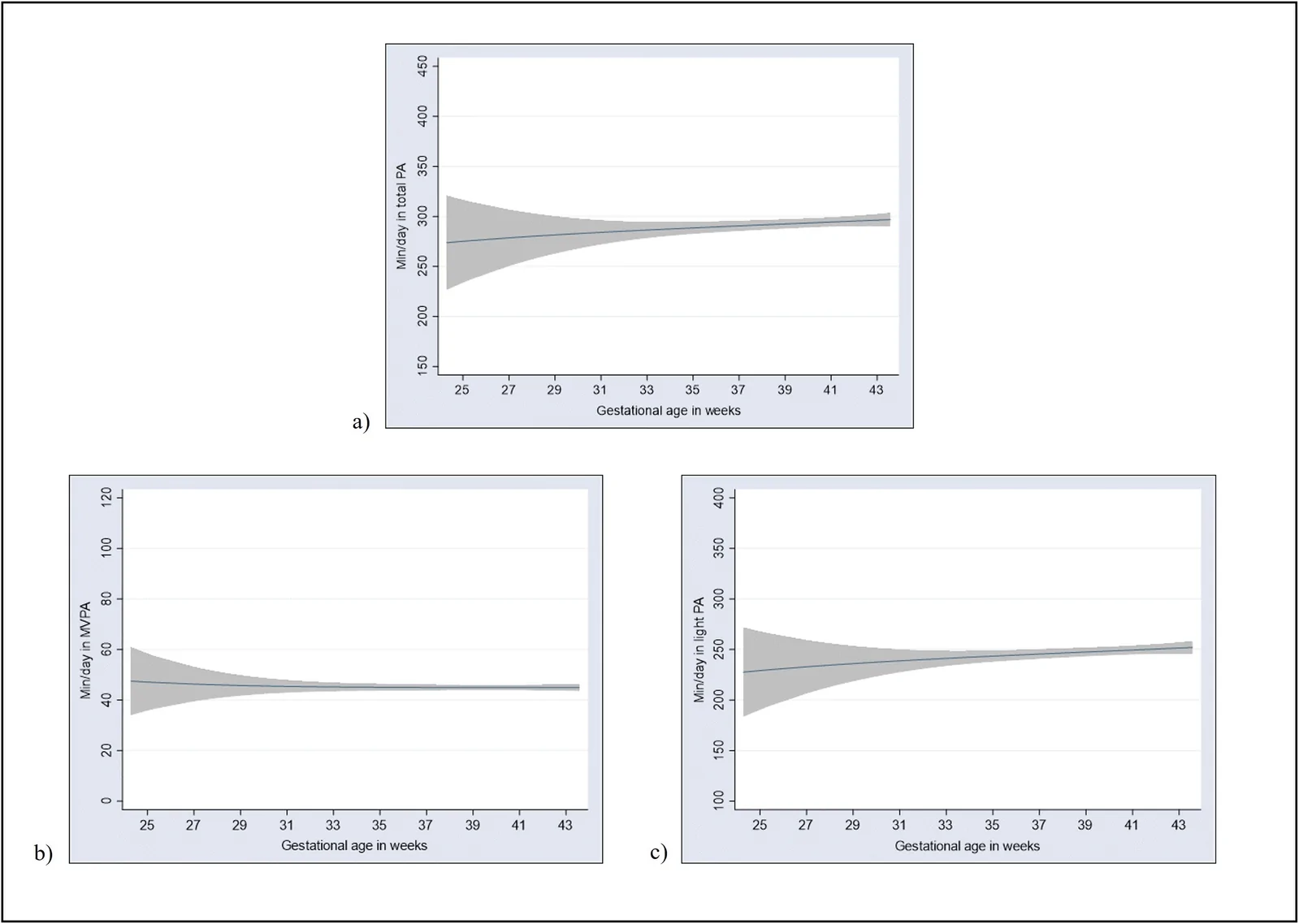
Min/day in PA categories by gestational age among adolescents and adults. The marked line shows min/day for **a)** total PA, **b)** MVPA and **c)** light PA from linear regression with fractional polynomials including 95% CI (shaded area), adjusted for age and sex (n = 12 896). Abbreviations: CI=Confidence interval; MVPA=moderate to vigorous physical activity; PA=physical activity.

Gestational age in weeks did not predict MET min/day in total PA, MVPA or light PA (Fig 2), or min/day in total PA, MVPA or light PA (Fig 3) in the non-linear model with fractional polynomials. The results were similar in separate analyses of females and males, and in Young-HUNT4 and HUNT4.

### Linear effects

In the linear model, gestational age in weeks predicted MET min/day in light PA (regression coefficient B = 1.53, 95% CI: 0.02 to 3.07, p = 0.039) in the total sample, and especially among females (B = 2.14, 95% CI: 0.33 to 3.93, p = 0.023) as shown in Table 3. In separate analyses of adolescents and adults, gestational age predicted MET min/day in total PA (B = 2.44, 95% CI: 0.16 to 4.88, p = 0.051) among adolescents.

**Table 3:** Linear association of metabolic equivalent of task (MET) min/day of physical activity categories by increase in gestational age in weeks among adolescents and adults.

Total sample	n = 12 896			Females n = 7752			Males n = 5144		
	**B**	(**95% CI)^a^**	p-value	**B**	**(95% CI)^b^**	p-value	**B**	**(95% CI)^b^**	p-value
Total PA	1.47	(−0.32 to 3.33)	0.101	1.31	(−0.87 to 3.51)	0.247	1.74	(−1.10 to 4.64)	0.242
MVPA	−0.06	(−0.95 to 0.85)	0.878	−0.83	(−1.87 to 0.20)	0.117	0.95	(−0.33 to 2.22)	0.143
Light PA	**1.53**	**(0.02 to 3.07)**	0.039	**2.14**	**(0.33 to 3.93)**	0.023	0.78	(−1.50 to 3.11)	0.512
**Young-HUNT4**	n = 4104			Females n = 2469			Males n = 1635		
	**B**	**(95% CI)^a^**	p-value	**B**	**(95% CI)^b^**	p-value	**B**	**(95% CI)^b^**	p-value
Total PA	**2.44**	**(0.16 to 4.88)**	0.051	2.00	(−0.88 to 4.77)	0.207	3.12	(−0.99 to 7.27)	0.146
MVPA	0.85	(−0.36 to 2.06)	0.184	0.19	(−1.40 to 1.73)	0.817	1.74	(−0.32 to 3.85)	0.108
Light PA	1.59	(−0.002 to 3.19)	0.066	1.81	(−0.23 to 3.65)	0.080	1.38	(−1.49 to 4.25)	0.352
**HUNT4**	n = 8792			Females n = 5283			Males n = 3509		
	**B**	**(95% CI)^a^**	p-value	**B**	**(95% CI)^b^**	p-value	**B**	**(95% CI)^b^**	p-value
Total PA	0.04	(−2.36 to 2.43)	0.977	−0.19	(−3.15 to 2.79)	0.898	0.37	(−3.17 to 3.89)	0.857
MVPA	0.22	(−0.83 to 1.19)	0.662	−0.37	(−1.68 to 0.90)	0.582	1.06	(−0.57 to 2.61)	0.149
Light PA	−0.18	(−2.15 to 1.67)	0.849	0.18	(−2.27 to 2.71)	0.882	−0.69	(−3.69 to 2.14)	0.646

Abbreviations: CI = confidence interval; HUNT4 = Trøndelag Health Study 2017–2019, Survey 4; MET = metabolic equivalent of task; MVPA = moderate to vigorous physical activity; PA = physical activity; Young-HUNT4 = Trøndelag Health Study 2017–2019 (13–19 years), Survey 4.

^a^Based on bootstrapped regression analysis with gestational age as independent variable, adjusted for age and sex.

^b^Based on bootstrapped regression analysis with gestational age as independent variable, adjusted for age.

Estimates with 95% confidence intervals not including zero are presented in bold.

Gestational age in weeks predicted min/day in total PA by 0.99 (95% CI: 0.07 to 1.90, p = 0.024) in the total sample, and by 1.25 (95% CI: 0.11 to 2.40, p = 0.027) among females (Table 4). Additionally, gestational age predicted min/day in light PA (B = 1.01, 95% CI: 0.16 to 1.89, p = 0.017) in the total sample, and among females (B = 1.43, 95% CI: 0.34 to 2.48, p = 0.009). In separate analyses of adolescents and adults, gestational age predicted min/day in total PA (B = 1.06, 95% CI: 0.02 to 2.12) among adolescents, although the corresponding p-value was slightly above 0.05 (p = 0.064).

**Table 4:** Linear association of min/day of physical activity categories by increase in gestational age in weeks among adolescents and adults.

Total sample	n = 12 896			Females n = 7752			Males n = 5144		
	**B**	**(95% CI)^a^**	p-value	**B**	**(95% CI)^b^**	p-value	**B**	**(95% CI)^b^**	p-value
Total PA	**0.99**	**(0.07 to 1.90)**	0.024	**1.25**	**(0.11 to 2.40)**	0.027	0.68	(−0.66 to 2.07)	0.347
MVPA	−0.03	(−0.22 to 0.18)	0.780	−0.18	(−0.42 to 0.05)	0.125	0.18	(−0.10 to 0.47)	0.215
Light PA	**1.01**	**(0.16 to 1.89)**	0.017	**1.43**	**(0.34 to 2.48)**	0.009	0.50	(−0.79 to 1.78)	0.460
**Young-HUNT4**	n = 4104			Females n = 2469			Males n = 1635		
	**B**	**(95% CI)^a^**	p-value	**B**	**(95% CI)^b^**	p-value	**B**	**(95% CI)^b^**	p-value
Total PA	**1.06**	**(0.02 to 2.12)**	0.064	1.13	(−0.16 to 2.36)	0.108	1.03	(−0.74 to 2.87)	0.280
MVPA	0.19	(−0.09 to 0.47)	0.195	0.06	(−0.31 to 0.40)	0.751	0.37	(−0.10 to 0.85)	0.126
Light PA	0.87	(−0.06 to 1.82)	0.089	1.07	(−0.09 to 2.15)	0.078	0.66	(−0.99 to 2.35)	0.423
**HUNT4**	n = 8792			Females n = 5283			Males n = 3509		
	**B**	**(95% CI)^a^**	p-value	**B**	**(95% CI)^b^**	p-value	**B**	**(95% CI)^b^**	p-value
Total PA	0.11	(−1.09 to 1.25)	0.853	0.25	(−1.17 to 1.69)	0.732	−0.09	(−1.89 to 1.65)	0.912
MVPA	0.03	(−0.21 to 0.25)	0.793	−0.09	(−0.40 to 0.21)	0.564	0.20	(−0.17 to 0.56)	0.280
Light PA	0.08	(−1.05 to 1.14)	0.883	0.34	(−1.03 to 1.75)	0.637	−0.29	(−1.98 to 1.35)	0.732

Abbreviations: CI = confidence interval; HUNT4 = Trøndelag Health Study 2017–2019, Survey 4; MVPA = moderate to vigorous physical activity; PA = physical activity; Young-HUNT4 = Trøndelag Health Study 2017–2019 (13–19 years), Survey 4.

^a^Based on bootstrapped regression analysis with gestational age as independent variable, adjusted for age and sex.

^b^Based on bootstrapped regression analysis with gestational age as independent variable, adjusted for age.

Estimates with 95% confidence intervals not including zero are presented in bold.

### Supplementary analyses

Among participants with valid accelerometry, the proportions born very and moderately preterm were 15–17% higher and the proportion born late preterm was 17% lower than among non-participants who did not wear accelerometers (S2 Table). In the term-born groups, the proportion was only 1–6% higher among participants than non-participants (S3 Table). Among adolescents born very preterm, a higher proportion took part in organized training, while a higher proportion of non-participants took part in non-organized training (S4 Table). In adults, a higher proportion of the participants had higher educational levels across all gestational age groups, and a lower proportion had long-term illness, except in the very and moderate preterm groups (S6-S7 Table). Slightly more adult participants than non-participants reported that they exercised once a week or more, and for 30 min or more, except in the moderately preterm group.

Birth weight did not predict MET min/day in total PA, MVPA or light PA (S1 Fig, S8 Table), or min/day in total PA, MVPA or light PA (S2 Fig, S9 Table) in the total sample or in separate analyses of adolescents and adults or among females or males in non-linear or linear models.

Linear associations between PA categories and gestational age in weeks, with additional adjustment for BMI, did not substantially change the results. Gestational age in weeks predicted MET min/day in total PA by 1.80 (95% CI: 0.03 to 3.61, p = 0.040) in the total sample, and by 2.56 (95% CI: 0.01 to 5.11, p = 0.044) in separate analyses of adults (S10 Table). Also, gestational age in weeks predicted MET min/day in light PA by 1.67 (95% CI: 0.23 to 3.13, p = 0.020) in the total sample, and among females (B = 2.33, 95% CI: 0.59 to 4.10, p = 0.011). Additionally, gestational age predicted min/day in total PA by 1.08 (95% CI: 0.21 to 1.96, p = 0.014) in the total sample, and by 1.42 (95% CI: 0.38 to 2.51, p = 0.009) among females (S11 Table). Also, gestational age predicted min/day in light PA by 1.07 (95% CI: 0.27 to 1.88, p = 0.011) in the total sample and by 1.53 (95% CI: 0.56 to 2.53, p = 0.002) among females. In separate analyses of adolescents and adults, gestational age predicted min/day in total PA (B = 1.10, 95% CI: 0.03 to 2.22, p = 0.054) among adults.

## Discussion

In this population-based register linkage study in Norway, there was a linear association between shorter gestation and less PA in the total sample, among females, and in adolescents. A strength of the study is the large population-based cohort with linkage to the MBRN, making it possible to examine device-measured PA across the range of gestational age in adolescents and adults from 13–52 years of age. In the fourth wave of the HUNT Study, participants from the northern region of the Trøndelag County were invited to wear tri-axial accelerometers. This region does not include a larger city and has a smaller proportion of immigrants to be fully representative for the Norwegian population [20]. However, as the HUNT Study has a high participation rate, the number of participants included in our study was considerably high. The large sample size allowed for separate analyses of females and males, and adolescents and adults.

Although the global prevalence of preterm birth is about 10% [1,2], the proportion in Norway is lower, at around 5.4% [33]. In our sample of 12 896 participants, 4.8% were born preterm, which is slightly lower than the expected national prevalence. Except for the late preterm and late term groups, more participants reported being moderate to vigorous physically active when exercising compared with non-participants. Thus, we cannot rule out the possibility of selection bias.

We used a machine learning model to predict PA behaviors with high accuracy [27–29]. Being able to classify key PA behaviors, such as running, cycling, brisk walking, moderate walking, slow walking and standing gives us unique insight into how PA takes place in everyday life for a substantial number of participants. Furthermore, combining MET min/day of these behaviors into total PA, MVPA and light PA, provides a much more precise measure of PA in everyday life compared to leisure-time exercise obtained by self-report. In addition, we presented PA in min/day to better enable comparisons with previous studies.

In a meta-analysis of 13 Nordic cohorts, the lower and upper ends of birth weight were associated with less leisure-time PA measured by self-report [9]. These associations were dependent on age, sex, gestational age, educational level, BMI and smoking. In our study, we adjusted for age and sex and conducted separate analyses for adolescents and adults, and females and males in the non-linear models. As no non-linear associations were found between gestational age or birth weight in total PA, MVPA, or light PA after adjusting for age and sex, additional adjustments for educational level, BMI and smoking were not considered necessary. However, in supplementary analyses of the linear associations between PA categories and gestational age in weeks, additional adjustment for BMI showed that the main results remained robust.

We found an association across the range of gestational age and min/day in total PA in the total sample. In a joint study of the HeSVA and NTNU LBW Life cohorts [19], the mean difference was −7.3 (95% CI: −13.9 to −1.0) min/day in MVPA and −16.9 (95% CI: −45.6 to 11.7) min/day in light PA between the VLBW group (mean gestational age of 29 weeks) and control group (mean gestational age of 40 weeks) when adjusted for cohort, age and sex. Even though the method for measuring PA was similar as in the present study, categories of PA and MET values differed slightly. In our study, the comparable min/day in total PA for a participant born very preterm at 29 weeks, when adjusted for age and sex, would be 283 min/day. For a term-born participant at 40 weeks, this would be 294 min/day in total PA. This corresponds to a mean difference of about −11 min/day in total PA, which is a smaller difference. However, when accumulated over a week or a month, this represents a considerable difference.

Comparison of absolute values of PA across studies is limited by methodological differences. Studies using device-measured PA differ substantially with respect to accelerometer type, placement, number of devices, and criteria of recording. In our study, participants wore two accelerometers on the thigh and back for seven days, with recording for >23 hours/day for at least two weekdays and on weekend day, to be included. Other studies have typically used a single device with shorter wear-time criteria (e.g., 8–10 hours/day) [16–18], which makes direct comparisons challenging.

Even though we demonstrated an association between gestational age and total PA, in particular light PA, we did not find that gestational age predicted MET min/day or min/day in MVPA. This is in line with a previous study of the HeSVA cohort of younger adults born preterm with VLBW compared with term-born controls at 25 years of age [17], and a study of adults born early term and late preterm compared with controls [18], where no difference was found in min/day of MVPA assessed by hip-worn and wrist-worn accelerometry.

The stronger association between gestational age and min/day in total PA and light PA in females may be consistent with a IPD meta-analysis showing a larger effect of being born very preterm or with VLBW on PA in women than in men, even though PA was measured by self-report [10]. Although other studies using accelerometry do not support the larger effect of preterm birth or birth weight on PA in women [17,18], individuals born preterm face an increased risk of adverse health outcomes [3–7]. As PA is associated with a reduced risk of cardiovascular disease [8,34,35], increasing PA in everyday life may be relevant for individuals born at lower gestational ages, especially females.

In this study, gestational age but not birth weight, was associated with min/day in total PA and light PA. Even though there was no linear association with MVPA, unadjusted mean values of min/day of MVPA indicated that all the gestational age groups had at least 35 min/day of MVPA, adding up to meet the adult recommendations of PA by the World Health Organization (WHO) of 150–300 min moderate PA, 75–150 min vigorous PA, or a combination of both per week [36]. However, when applying the WHO recommendations for children (aged 5–17 years) among the adolescents aged 13–19 years, none of the adolescent groups in our study met the recommendation of at least 60 min/day of MVPA, corresponding to ≥420 min per week [36]. This suggests that the adolescents born preterm might benefit from being more physically active, and that the time spent in PA among adolescents may be overestimated when interpreted using adult-based thresholds.

As highlighted in the 2020 WHO guidelines, *every move count* and *more is better* [37], which means that even small increases in everyday PA may provide health benefits [8]. Promoting PA from an early age may therefore be key to establishing lifelong PA habits, particularly among individuals born preterm.

## Conclusion

In conclusion, there was a positive linear association between gestational age in weeks and MET min/day in light PA in this large population-based register linkage study of adolescents and adults. The association was stronger among females. There was also an association between gestational age and MET min/day in total PA among adolescents. Similar associations were found with min/day in light PA in the total sample and with min/day in total PA in the total sample, among females and among adolescents. There were no non-linear or linear effects of birth weight on PA. Although there were no associations between gestational age and MVPA, the associations with total PA are by and large consistent with the previous observations that individuals born preterm with VLBW have lower rates of PA later in life.

## Supporting information

S1 Fig Metabolic equivalent of task (MET) min/day in physical activity categories by birth weight in 100 grams among adolescents and adults. The marked line shows MET min/day for **a)** total PA, **b)** MVPA and **c)** light PA from linear regression with fractional polynomials including 95% CI (shaded area), adjusted for age and sex (n = 12 896). Abbreviations: CI: Confidence interval; MET = metabolic equivalent of task; MVPA = moderate to vigorous physical activity; PA = physical activity.
(TIF)

S2 Fig Min/day in physical activity categories by birth weight in 100 grams among adolescents and adults. The marked line shows min/day for **a)** total PA, **b)** MVPA and **c)** light PA from linear regression with fractional polynomials including 95% CI (shaded area), adjusted for age and sex (n = 12 896). Abbreviations: CI: Confidence interval; MVPA = moderate to vigorous physical activity; PA = physical activity.
(TIF)

S1 Table Conversion factor of metabolic equivalent of task (MET) minutes of physical activity behaviors among adolescents and adults. Abbreviations: MET = metabolic equivalent of task; MVPA = moderate to vigorous physical activity; PA = physical activity. ^a^Running: 6.4 km/h equals lower limit for jogging. ^b^Brisk walking: 5.5 km/h equals lower limit for brisk walking. ^c^Moderate walking: 4.1–5.4 km/h equals range for moderate walking. ^d^Slow walking: 4 km/h equals upper limit for slow walking.
(DOCX)

S2 Table Background characteristics of study participants and non-participants by preterm gestational age group. Abbreviation: SD = standard deviation.
(DOCX)

S3 Table Background characteristics of study participants and non-participants by term gestational age group. Abbreviation: SD = standard deviation.
(DOCX)

S4 Table Background data of study participants and non-participants in Young-HUNT4 by preterm gestational age group. *Exact number not shown for counts below 5 due to privacy reasons. Abbreviations: Young-HUNT4 = Trøndelag Health Study 2017–2019 (13–19 years), Survey 4. ^a^Data missing for two very preterm participants, two very preterm non-participants, one moderately preterm participant, one moderately preterm non-participant, ten late preterm participants and six late preterm non-participants. ^b^Data missing for two very preterm participants, four late preterm participants and three late preterm non-participants. ^c^Data missing for three very preterm participants, one very preterm non-participant, two moderately preterm participants, ten late preterm participants and five late preterm non-participants. ^d^Data missing for one very preterm participant, one moderately preterm participant, six late preterm participants and three late preterm non-participants. ^e^Data missing for two very preterm participants, one moderately preterm participant, five late preterm participants and three late preterm non-participants. ^f^Data missing for two very preterm participants, three moderately preterm participants, seven late preterm participants and four late preterm non-participants. ^g^Data missing for two very preterm participants, one moderately preterm participant, five late preterm participants and three late preterm non-participants.
(DOCX)

S5 Table Background data of study participants and non-participants in Young-HUNT4 by term gestational age group. *Exact number not shown for counts below 5 due to privacy reasons. Abbreviations: Young-HUNT4 = Trøndelag Health Study 2017–2019 (13–19 years), Survey 4. ^a^Data missing for 24 early term participants, 18 early term non-participants, 60 full term participants, 56 full term non-participants, 28 late term participants, 19 late term nonparticipants, eight post term participants and nine post term non-participants. ^b^Data missing for twelve early term participants, ten early term non-participants, 25 full term participants, 27 full term non-participants, 9 late term participants, 7 late term non-participants, one post term participant and seven post term non-participants. ^c^Data missing for 28 early term participants, 17 early term non-participants, 51 full term participants, 51 full term non-participants, 30 late term participants, 18 late term nonparticipants, 6 post term participants and 9 post term non-participants. ^d^Data missing for twelve early term participants, 14 early term non-participants, 34 full term participants, 31 full term non-participants, 13 late term participants, 16 late term non-participants, two post term participants and three post term non-participants. ^e^Data missing for 19 early term participants, 20 early term non-participants, 38 full term participants, 42 full term non-participants, 12 late term participants, 19 late term nonparticipants, three post term participants and one post term non-participant. ^f^Data missing for 21 early term participants, 21 early term non-participants, 37 full term participants, 38 full term non-participants, 17 late term participants, 17 late term nonparticipants, six post term participants and two post term non-participants. ^g^Data missing for 16 early term participants, 15 early term non-participants, 35 full term participants, 34 full term non-participants, twelve late term participants, 15 late term non-participants, four post term participants and two post term non-participants.
(DOCX)

S6 Table Background data of study participants and non-participants in HUNT4 by preterm gestational age group. *Exact number not shown for counts below 5 due to privacy reasons. Abbreviations: HUNT4 = Trøndelag Health Study 2017–2019, Survey 4. ^a^Data missing for one moderately preterm non-participant and one late preterm non-participant. ^b^Data missing for one late preterm participant and two late preterm non-participants. ^c^Data missing for one very preterm participant, one very preterm non-participant, one moderately preterm participant, two moderately preterm non-participants, five late preterm participants and five late preterm non-participants. ^d^Data missing for five very preterm participants, nine very preterm non-participants, seven moderately preterm participants, 17 moderately preterm non-participants, 43 late preterm participants and 89 late preterm non-participants. ^e^Data missing for five very preterm participants, ten very preterm non-participants, seven moderately preterm participants, 17 moderately preterm non-participants, 42 late preterm participants and 88 late preterm non-participants.
(DOCX)

S7 Table Background data of study participants and non-participants in HUNT4 by term gestational age group. Abbreviations: HUNT4 = Trøndelag Health Study 2017–2019, Survey 4. ^a^Data missing for one early term participant, ten early term non-participants, ten full term participants, 33 full term non-participants, seven late term participants, 11 late term non-participants, three post term participants and four post term non-participants. ^b^Data missing for eight early term participants, ten early term non-participants, 31 full term participants, 26 full term non-participants, 16 late term participants, 20 late term non-participants, eight post term participants and six post term non-participants. ^c^Data missing for five early term participants, 19 early term non-participants, 41 full term participants, 54 full term non-participants, 18 late term participants, 36 late term non-participants, 13 post term participants and 8 post term non-participants. ^d^Data missing for 195 early term participants, 251 early term non-participants, 738 full term participants, 970 full term non-participants, 357 late term participants, 471 late term non-participants, 214 post term participants and 286 post term non-participants. ^e^Data missing for 192 early term participants, 250 early term non-participants, 732 full term participants, 963 full term non-participants, 355 late term participants, 468 late term non-participants and 208 post term participants and 284 post term non-participants.
(DOCX)

S8 Table Linear association of metabolic equivalent of task (MET) min/day of physical activity categories by 100 grams increase in birth weight among adolescents and adults. Abbreviations: CI = confidence interval; HUNT4 = Trøndelag Health Study 2017–2019, Survey 4; MET = metabolic equivalent of task; MVPA = moderate to vigorous physical activity; PA = physical activity; Young-HUNT4 = Trøndelag Health Study 2017–2019 (13–19 years), Survey 4. ^a^Based on bootstrapped regression analysis with birth weight as independent variable, adjusted for age and sex. ^b^Based on bootstrapped regression analysis with birth weight as independent variable, adjusted for age.
(DOCX)

S9 Table Linear association of min/day of physical activity categories by 100 grams increase in birth weight among adolescents and adults. Abbreviations: CI = confidence interval; HUNT4 = Trøndelag Health Study 2017–2019, Survey 4; MVPA = moderate to vigorous physical activity; PA = physical activity; Young-HUNT4 = Trøndelag Health Study 2017–2019 (13–19 years), Survey 4. ^a^Based on bootstrapped regression analysis with birth weight as independent variable, adjusted for age and sex. ^b^Based on bootstrapped regression analysis with birth weight as independent variable, adjusted for age.
(DOCX)

S10 Table Linear association of metabolic equivalent of task (MET) min/day of physical activity categories by increase in gestational age in weeks among adolescents and adults with additional adjustment for body mass index. *Participants with available data on age, sex and body mass index (kg/m^2^). Abbreviations: CI = confidence interval; HUNT4 = Trøndelag Health Study 2017–2019, Survey 4; MET = metabolic equivalent of task; MVPA = moderate to vigorous physical activity; PA = physical activity; Young-HUNT4 = Trøndelag Health Study 2017–2019 (13–19 years), Survey 4. ^a^Based on bootstrapped regression analysis with gestational age as independent variable, adjusted for age, sex and body mass index. ^b^Based on bootstrapped regression analysis with gestational age as independent variable, adjusted for age and body mass index. Estimates with 95% confidence intervals not including zero are presented in bold.
(DOCX)

S11 Table Linear association of min/day of physical activity categories by increase in gestational age in weeks among adolescents and adults with additional adjustment for body mass index. *Participants with available data on age, sex and body mass index (kg/m^2^). Abbreviations: CI = confidence interval; HUNT4 = Trøndelag Health Study 2017–2019, Survey 4; MVPA = moderate to vigorous physical activity; PA = physical activity; Young-HUNT4 = Trøndelag Health Study 2017–2019 (13–19 years), Survey 4. ^a^Based on bootstrapped regression analysis with gestational age as independent variable, adjusted for age, sex and body mass index. ^b^Based on bootstrapped regression analysis with gestational age as independent variable, adjusted for age and body mass index. Estimates with 95% confidence intervals not including zero are presented in bold.
(DOCX)

## Abbreviations

BMI: Body mass index (kg/m^2^)
CI: Confidence interval
ESTER: ESTER Preterm Birth Study
HeSVA: Helsinki Study of Very Low Birth Weight Adults
HUNT: Trøndelag Health Study
HUNT4: Trøndelag Health Study 2017–2019, Survey 4
IPD: Individual participant data
MBRN: Medical Birth Registry of Norway
MET: Metabolic equivalent of task
MVPA: Moderate to vigorous physical activity
NTNU LBW Life: Norwegian University of Science and Technology Low Birth Weight in a Lifetime Perspective study
PA: Physical activity
REK: Regional Committee for Medical and Health Research Ethics
SD: Standard deviation
VLBW: Very low birth weight (<1500g)
XGBoost: eXtreme Gradient Boosting
Young-HUNT4: Trøndelag Health Study 2017–2019 (13–19 years), Survey 4

## References

[1] Ohuma EO, Moller A-B, Bradley E, Chakwera S, Hussain-Alkhateeb L, Lewin A, et al. National, regional, and global estimates of preterm birth in 2020, with trends from 2010: a systematic analysis. Lancet. 2023;402(10409):1261–71. doi: 10.1016/S0140-6736(23)00878-4 37805217

[2] Lawn JE, Ohuma EO, Bradley E, Idueta LS, Hazel E, Okwaraji YB, et al. Small babies, big risks: global estimates of prevalence and mortality for vulnerable newborns to accelerate change and improve counting. Lancet. 2023;401(10389):1707–19. doi: 10.1016/S0140-6736(23)00522-6 37167989

[3] Moster D, Lie RT, Markestad T. Long-term medical and social consequences of preterm birth. N Engl J Med. 2008;359(3):262–73. doi: 10.1056/NEJMoa0706475 18635431

[4] Kajantie E, Hovi P. Is very preterm birth a risk factor for adult cardiometabolic disease?. Semin Fetal Neonatal Med. 2014;19(2):112–7. doi: 10.1016/j.siny.2013.11.006 24332842

[5] Doyle LW, Andersson S, Bush A, Cheong JLY, Clemm H, Evensen KAI, et al. Expiratory airflow in late adolescence and early adulthood in individuals born very preterm or with very low birthweight compared with controls born at term or with normal birthweight: a meta-analysis of individual participant data. Lancet Respir Med. 2019;7(8):677–86. doi: 10.1016/S2213-2600(18)30530-7 31078498

[6] Pyhälä R, Wolford E, Kautiainen H, Andersson S, Bartmann P, Baumann N, et al. Self-reported mental health problems among adults born preterm: a meta-analysis. Pediatrics. 2017;139(4):e20162690. doi: 10.1542/peds.2016-2690 28283612

[7] Anderson PJ, de Miranda DM, Albuquerque MR, Indredavik MS, Evensen KAI, Van Lieshout R, et al. Psychiatric disorders in individuals born very preterm / very low-birth weight: An individual participant data (IPD) meta-analysis. EClinicalMedicine. 2021;42:101216. doi: 10.1016/j.eclinm.2021.101216 34901794 PMC8639417

[8] Warburton DER, Bredin SSD. Health benefits of physical activity: a systematic review of current systematic reviews. Curr Opin Cardiol. 2017;32(5):541–56. doi: 10.1097/HCO.0000000000000437 28708630

[9] Andersen LG, Angquist L, Gamborg M, Byberg L, Bengtsson C, Canoy D, et al. Birth weight in relation to leisure time physical activity in adolescence and adulthood: meta-analysis of results from 13 nordic cohorts. PLoS One. 2009;4(12):e8192. doi: 10.1371/journal.pone.0008192 20016780 PMC2790716

[10] Aakvik KAD, Benum SD, Tikanmäki M, Hovi P, Räikkönen K, Harris SL, et al. Physical activity and cognitive function in adults born very preterm or with very low birth weight-an individual participant data meta-analysis. PLoS One. 2024;19(2):e0298311. doi: 10.1371/journal.pone.0298311 38349926 PMC10863878

[11] Kaseva N, Wehkalampi K, Strang-Karlsson S, Salonen M, Pesonen A-K, Räikkönen K, et al. Lower conditioning leisure-time physical activity in young adults born preterm at very low birth weight. PLoS One. 2012;7(2):e32430. doi: 10.1371/journal.pone.0032430 22384247 PMC3288099

[12] Kajantie E, Strang-Karlsson S, Hovi P, Räikkönen K, Pesonen A-K, Heinonen K, et al. Adults born at very low birth weight exercise less than their peers born at term. J Pediatr. 2010;157(4):610–6, 616.e1. doi: 10.1016/j.jpeds.2010.04.002 20493499

[13] Yang J, Epton MJ, Harris SL, Horwood J, Kingsford RA, Troughton R, et al. Reduced exercise capacity in adults born at very low birth weight: a population-based cohort study. Am J Respir Crit Care Med. 2022;205(1):88–98. doi: 10.1164/rccm.202103-0755OC 34499592

[14] Vrijlandt EJLE, Gerritsen J, Boezen HM, Grevink RG, Duiverman EJ. Lung function and exercise capacity in young adults born prematurely. Am J Respir Crit Care Med. 2006;173(8):890–6. doi: 10.1164/rccm.200507-1140OC 16456146

[15] Tikanmäki M, Kaseva N, Tammelin T, Sipola-Leppänen M, Matinolli H-M, Eriksson JG, et al. Leisure time physical activity in young adults born preterm. J Pediatr. 2017;189:135-142.e2. doi: 10.1016/j.jpeds.2017.06.068 28751124

[16] Morrison KM, Gunn E, Guay S, Obeid J, Schmidt LA, Saigal S. Grip strength is lower in adults born with extremely low birth weight compared to term-born controls. Pediatr Res. 2021;89(4):996–1003. doi: 10.1038/s41390-020-1012-5 32555537

[17] Kaseva N, Martikainen S, Tammelin T, Hovi P, Järvenpää A-L, Andersson S, et al. Objectively measured physical activity in young adults born preterm at very low birth weight. J Pediatr. 2015;166(2):474–6. doi: 10.1016/j.jpeds.2014.10.018 25454929

[18] Tikanmäki M, Tammelin T, Kaseva N, Sipola-Leppänen M, Matinolli H-M, Hakonen H, et al. Objectively measured physical activity and sedentary time in young adults born preterm-The ESTER study. Pediatr Res. 2017;81(4):550–5. doi: 10.1038/pr.2016.262 27935902

[19] Benum SD, Aakvik KAD, Mehl CV, Kongsvold A, Lydersen S, Vollsæter M, et al. Device-measured physical activity in adults born preterm with very low birth weight and mediation by motor abilities. PLoS One. 2025;20(1):e0312875. doi: 10.1371/journal.pone.0312875 39775188 PMC11706474

[20] Åsvold BO, Langhammer A, Rehn TA, Kjelvik G, Grøntvedt TV, Sørgjerd EP, et al. Cohort profile update: the HUNT study, Norway. Int J Epidemiol. 2023;52(1):e80–91. doi: 10.1093/ije/dyac095 35578897 PMC9908054

[21] Rangul V, Holmen TL, Langhammer A, Ingul JM, Pape K, Fenstad JS, et al. Cohort profile update: the young-HUNT study, Norway. Int J Epidemiol. 2024;53. doi: 10.1093/ije/dyae013 38302751 PMC10834360

[22] Bakken IJ, Ariansen AMS, Knudsen GP, Johansen KI, Vollset SE. The Norwegian Patient Registry and the Norwegian Registry for Primary Health Care: Research potential of two nationwide health-care registries. Scand J Public Health. 2020;48(1):49–55. doi: 10.1177/1403494819859737 31288711

[23] Skjaerven R, Gjessing HK, Bakketeig LS. Birthweight by gestational age in Norway. Acta Obstet Gynecol Scand. 2000;79(6):440–9. doi: 10.1034/j.1600-0412.2000.079006440.x 10857867

[24] Quinn J-A, Munoz FM, Gonik B, Frau L, Cutland C, Mallett-Moore T, et al. Preterm birth: Case definition & guidelines for data collection, analysis, and presentation of immunisation safety data. Vaccine. 2016;34(49):6047–56. doi: 10.1016/j.vaccine.2016.03.045 27743648 PMC5139808

[25] Kongsvold A, Flaaten M, Logacjov A, Skarpsno ES, Bach K, Nilsen TIL, et al. Can the bias of self-reported sitting time be corrected? a statistical model validation study based on data from 23 993 adults in the Norwegian HUNT study. Int J Behav Nutr Phys Act. 2023;20(1):139. doi: 10.1186/s12966-023-01541-y 38012746 PMC10680356

[26] Kongsvold A, Flaaten M, Skarpsno ES, Logacjov A, Bach K, Lund Nilsen TI, et al. Contribution of walking, running and cycling to moderate-to-vigorous and total physical activity in adolescents and adults across and within seasons: cross-sectional data from the Norwegian HUNT Study. BMJ Open Sport Exerc Med. 2025;11(4):e002394. doi: 10.1136/bmjsem-2024-002394 41181313 PMC12574398

[27] Logacjov A, Ludvigsen TP, Bach K, Kongsvold A, Flaaten M, Lund Nilsen TI, et al. The performance of a machine learning model in predicting accelerometer-derived walking speed. Heliyon. 2025;11(2):e42185. doi: 10.1016/j.heliyon.2025.e42185 39925351 PMC11804687

[28] Logacjov A, Bach K, Kongsvold A, Bårdstu HB, Mork PJ. HARTH: a human activity recognition dataset for machine learning. Sensors (Basel). 2021;21(23):7853. doi: 10.3390/s21237853 34883863 PMC8659926

[29] Bach K, Kongsvold A, Bårdstu H, Bardal EM, Kjærnli HS, Herland S, et al. A machine learning classifier for detection of physical activity types and postures during free-living. J Meas Phys Behav. 2022;5(1):24–31. doi: 10.1123/jmpb.2021-0015

[30] Herrmann SD, Willis EA, Ainsworth BE, Barreira TV, Hastert M, Kracht CL, et al. 2024 Adult compendium of physical activities: a third update of the energy costs of human activities. J Sport Health Sci. 2024;13(1):6–12. doi: 10.1016/j.jshs.2023.10.010 38242596 PMC10818145

[31] Mitchell MN. Interpreting and Visualizing Regression Models Using Stata. Texas: Stata Press. 2021.

[32] Spiegler J, Eves R, Mendonça M, Wolke D. Association of physical activity and cardiorespiratory function or BMI and body composition in preterm-born individuals: a systematic review. Acta Paediatr. 2019;108(7):1205–14. doi: 10.1111/apa.14726 30664798

[33] Thomsen L, Daltveit A, Næss T, Juliusson P, Engjom H, Akerkar R. Årsrapport for Medisinsk fødselsregister 2024. Svangerskap og fødsler i Norge [Annual report from the Medical Birth Registry of Norway 2024. Pregnancy and births in Norway]. Rapport 2024. Oslo: Folkehelseinstituttet. 2025.

[34] Liang Z, Zhang M, Wang C-Z, Yuan Y, Liang J-H. Association between sedentary behavior, physical activity, and cardiovascular disease-related outcomes in adults-A meta-analysis and systematic review. Front Public Health. 2022;10:1018460. doi: 10.3389/fpubh.2022.1018460 36339165 PMC9632849

[35] Zhao M, Veeranki SP, Li S, Steffen LM, Xi B. Beneficial associations of low and large doses of leisure time physical activity with all-cause, cardiovascular disease and cancer mortality: a national cohort study of 88,140 US adults. Br J Sports Med. 2019;53(22):1405–11. doi: 10.1136/bjsports-2018-099254 30890520

[36] Bull FC, Al-Ansari SS, Biddle S, Borodulin K, Buman MP, Cardon G, et al. World Health Organization 2020 guidelines on physical activity and sedentary behaviour. Br J Sports Med. 2020;54(24):1451–62. doi: 10.1136/bjsports-2020-102955 33239350 PMC7719906

[37] World Health Organization. WHO guidelines on physical activity and sedentary behaviour: at a glance. Geneva: World Health Organization, 2020.

