# Supplementary material for: Gestational age and device-measured physical activity in adolescents and adults: A linkage of the HUNT Study and Medical Birth Registry
Source: PLoS One. 2026 Sep 23;21(9):e0357277. doi: 10.1371/journal.pone.0357277 (PMC13600527; doi:10.1371/journal.pone.0357277)
Supplement: Supplementary file 1 [file pone.0357277.s003.docx]

**S1 Table.** **Conversion factor of metabolic equivalent of task (MET) minutes of physical activity behaviors among adolescents and adults.**

| **PA category** | | **PA behavior** | **Compendium 2024** [1] | | | **Conversion factor MET min** |
| --- | --- | --- | --- | --- | --- | --- |
|  |  |  | **Code** | **Description** | **Estimated MET** |  |
| Total PA | MVPA | Running | 12028 | Running, 6.4 to 6.75 km/h (13 min/mile) | 6.5 | 6.0^a^ |
|  |  | Cycling | 01010 | Bicycling, <16 km/h, leisure, to work or for pleasure | 4.0 | 4.0 |
|  |  | Brisk walking | 17200 | Walking, 5.5 km/h, level, brisk, firm surface, walking for exercise | 4.8 | 4.5^b^ |
|  |  | Moderate walking | 17190 | Walking, 4.1 to 5.5 km/h, level, moderate pace, firm surface | 3.8 | 3.5^c^ |
|  | Light PA | Slow walking | 17170 | Walking, 4 km/h, level, firm surface | 3.0 | 2.8^d^ |
|  |  | Standing | 17041 | Standing, fidgeting | 1.5 | 1.5 |

Abbreviations: MET=metabolic equivalent of task; MVPA=moderate to vigorous physical activity; PA=physical activity.

^a^Running: 6.4 km/h equals lower limit for jogging.

^b^Brisk walking: 5.5 km/h equals lower limit for brisk walking.

^c^Moderate walking: 4.1-5.4 km/h equals range for moderate walking.

^d^Slow walking: 4 km/h equals upper limit for slow walking.

Reference

1.	Herrmann SD, Willis EA, Ainsworth BE, Barreira TV, Hastert M, Kracht CL, et al. 2024 Adult Compendium of Physical Activities: A third update of the energy costs of human activities. J Sport Health Sci. 2024;13(1):6–12. doi: 10.1016/j.jshs.2023.10.010. PubMed PMID: 38242596; PubMed Central PMCID: PMCPMC10818145.
