# Supplementary material for: Gestational age and device-measured physical activity in adolescents and adults: A linkage of the HUNT Study and Medical Birth Registry
Source: PLoS One. 2026 Sep 23;21(9):e0357277. doi: 10.1371/journal.pone.0357277 (PMC13600527; doi:10.1371/journal.pone.0357277)
Supplement: Supplementary file 2 [file pone.0357277.s004.docx]

**S2 Table.** **Background characteristics of study participants and non-participants by preterm gestational age group.**

|  | Participants and non-participants by preterm gestational age group (n=1291) | | | | | | | | | | | |
| --- | --- | --- | --- | --- | --- | --- | --- | --- | --- | --- | --- | --- |
|  | Very preterm (<32 weeks) | | | | Moderately preterm (32-33 weeks) | | | | Late preterm (34-36 weeks) | | | |
|  | Participants  (n=73) | | Non-participants  (n=62) | | Participants  (n=92) | | Non-participants  (n=76) | | Participants  (n=456) | | Non-participants  (n=532) | |
| Gestational age (weeks), mean (SD) | 29.5 | (1.8) | 29.4 | (2.0) | 33.0 | (0.5) | 33.1 | (0.6) | 35.8 | (0.8) | 35.8 | (0.8) |
| Birth weight z-scores, mean (SD) | -0.3 | (1.1) | -0.003 | (1.3) | -0.2 | (1.4) | -0.1 | (1.3) | -0.1 | (1.2) | -0.1 | (1.2) |
| Birth weight (g), mean (SD) | 1326 | (428) | 1386 | (490) | 2077 | (524) | 2146 | (481) | 2762 | (563) | 2754 | (543) |
| Female, n (%) | 44 | (60.3) | 33 | (53.2) | 53 | (57.6) | 31 | (40.8) | 260 | (57.0) | 260 | (48.9) |
| Birthyear in categories, n (%) |  |  |  |  |  |  |  |  |  |  |  |  |
| 1967-1976 | 15 | (20.5) | 17 | (27.4) | 23 | (25.0) | 23 | (30.3) | 101 | (22.1) | 147 | (27.6) |
| 1977-1986 | 7 | (9.6) | 12 | (19.4) | 7 | (7.6) | 13 | (17.1) | 78 | (17.1) | 98 | (18.4) |
| 1987-1996 | 11 | (15.1) | 10 | (16.1) | 12 | (13.0) | 15 | (19.7) | 81 | (17.8) | 130 | (24.4) |
| 1997-2005 | 40 | (54.8) | 23 | (37.1) | 50 | (54.3) | 25 | (32.9) | 196 | (43.0) | 157 | (29.5) |

Abbreviation: SD=standard deviation.
