# Supplementary material for: Gestational age and device-measured physical activity in adolescents and adults: A linkage of the HUNT Study and Medical Birth Registry
Source: PLoS One. 2026 Sep 23;21(9):e0357277. doi: 10.1371/journal.pone.0357277 (PMC13600527; doi:10.1371/journal.pone.0357277)
Supplement: Supplementary file 3 [file pone.0357277.s005.docx]

|  | Participants and non-participants by term gestational age group (n=24 020) | | | | | | | | | | | | | | | |
| --- | --- | --- | --- | --- | --- | --- | --- | --- | --- | --- | --- | --- | --- | --- | --- | --- |
|  | Early term (37-38 weeks) | | | | Full term (39-40 weeks) | | | | Late term (41 weeks) | | | | Post term (42-43.6 weeks) | | | |
|  | Participants  (n=1798) | | Non-participants  (n=1693) | | Participants  (n=6120) | | Non-participants  (n=5823) | | Participants  (n=2745) | | Non-participants  (n=2705) | | Participants  (n=1612) | | Non-participants  (n=1524) | |
| Gestational age (weeks), mean (SD) | 38.2 | (0.5) | 38.2 | (0.5) | 40.0 | (0.6) | 40.0 | (0.5) | 41.4 | (0.3) | 41.4 | (0.3) | 42.5 | (0.4) | 42.5 | (0.4) |
| Birth weight z-scores, mean (SD) | -0.1 | (1.1) | -0.1 | (1.1) | 0.02 | (1.0) | -0.1 | (1.0) | 0.04 | (1.0) | 0.02 | (1.0) | 0.02 | (1.0) | 0.06 | (1.0) |
| Birth weight (g), mean (SD) | 3269 | (496) | 3261 | (497) | 3592 | (464) | 3574 | (473) | 3737 | (479) | 3738 | (481) | 3760 | (476) | 3793 | (497) |
| Female, n (%) | 1063 | (59.1) | 802 | (47.4) | 3725 | (60.9) | 2923 | (50.2) | 1640 | (59.7) | 1395 | (51.6) | 968 | (60.0) | 800 | (52.5) |
| Birthyear in categories, n (%) |  |  |  |  |  |  |  |  |  |  |  |  |  |  |  |  |
| 1967-1976 | 458 | (25.5) | 509 | (30.1) | 1871 | (30.6) | 1878 | (32.3) | 883 | (32.2) | 863 | (31.9) | 547 | (33.9) | 547 | (35.9) |
| 1977-1986 | 266 | (14.8) | 281 | (16.6) | 1207 | (19.7) | 1262 | (21.7) | 594 | (21.6) | 650 | (24.0) | 409 | (25.4) | 388 | (25.5) |
| 1987-1996 | 238 | (13.2) | 353 | (20.9) | 925 | (15.1) | 1248 | (21.4) | 466 | (17.0) | 597 | (22.1) | 287 | (17.8) | 335 | (22.0) |
| 1997-2005 | 836 | (46.5) | 550 | (32.5) | 2117 | (34.6) | 1434 | (24.6) | 802 | (29.2) | 595 | (22.0) | 370 | (22.9) | 254 | (16.7) |

**S3 Table.** **Background characteristics of study participants and non-participants by term gestational age group.**

Abbreviation: SD=standard deviation.
