# Supplementary material for: Gestational age and device-measured physical activity in adolescents and adults: A linkage of the HUNT Study and Medical Birth Registry
Source: PLoS One. 2026 Sep 23;21(9):e0357277. doi: 10.1371/journal.pone.0357277 (PMC13600527; doi:10.1371/journal.pone.0357277)
Supplement: Supplementary file 4 [file pone.0357277.s006.docx]

**S4 Table.** **Background data of study participants and non-participants in Young-HUNT4 by preterm gestational age group.**

|  | Participants and non-participants by preterm gestational age group (n=438) | | | | | | | | | | | |
| --- | --- | --- | --- | --- | --- | --- | --- | --- | --- | --- | --- | --- |
|  | Very preterm (<32 weeks) | | | | Moderately preterm (32-33 weeks) | | | | Late preterm (34-36 weeks) | | | |
|  | Participants  (n=36) | | Non-participants  (n=20) | | Participants  (n=47) | | Non-participants  (n=23) | | Participants  (n=184) | | Non-participants  (n=128) | |
| Motor impairment (moderate/much)^a^, n (%) | * | * | * | * | * | * | * | * | * | * | * | * |
| Visual impairment (moderate/much)^b^, n (%) | 5 | (13.9) | * | * | 5 | (10.6) | * | * | 24 | (13.0) | 19 | (14.8) |
| Hearing impairment (moderate/much)^c^, n (%) | * | * | * | * | * | * | * | * | 7 | (3.8) | * | * |
| Outside school: Playing sports or exercise so that you get out of breath or sweat for 2 hours or more a week^d^, n (%) | 19 | (54.3) | 13 | (65.0) | 34 | (73.9) | 14 | (60.9) | 127 | (71.3) | 66 | (52.8) |
| Partake in organized training once a week or more^e^, n (%) | 19 | (55.9) | 8 | (40.0) | 32 | (69.6) | 13 | (56.5) | 85 | (47.5) | 48 | (38.4) |
| Partake in non-organized training with others once a week or more^f^, n (%) | 15 | (44.1) | 13 | (65.0) | 25 | (56.8) | 10 | (43.5) | 83 | (46.9) | 57 | (46.0) |
| Exercise on your own once a week or more^g^, n (%) | 21 | (61.8) | 14 | (70.0) | 28 | (60.9) | 12 | (52.2) | 100 | (55.9) | 73 | (58.4) |

*Exact number not shown for counts below 5 due to privacy reasons.

Abbreviation: Young-HUNT4=Trøndelag Health Study 2017-2019 (13-19 years), Survey 4.

^a^Data missing for two very preterm participants, two very preterm non-participants, one moderately preterm participant, one moderately preterm non-participant, ten late preterm participants and six late preterm non-participants.

^b^Data missing for two very preterm participants, four late preterm participants and three late preterm non-participants.

^c^Data missing for three very preterm participants, one very preterm non-participant, two moderately preterm participants, ten late preterm participants and five late preterm non-participants.

^d^Data missing for one very preterm participant, one moderately preterm participant, six late preterm participants and three late preterm non-participants.

^e^Data missing for two very preterm participants, one moderately preterm participant, five late preterm participants and three late preterm non-participants.

^f^Data missing for two very preterm participants, three moderately preterm participants, seven late preterm participants and four late preterm non-participants.

^g^Data missing for two very preterm participants, one moderately preterm participant, five late preterm participants and three late preterm non-participants.
