# Supplementary material for: Gestational age and device-measured physical activity in adolescents and adults: A linkage of the HUNT Study and Medical Birth Registry
Source: PLoS One. 2026 Sep 23;21(9):e0357277. doi: 10.1371/journal.pone.0357277 (PMC13600527; doi:10.1371/journal.pone.0357277)
Supplement: Supplementary file 5 [file pone.0357277.s007.docx]

**S5 Table.** **Background data of study participants and non-participants in Young-HUNT4 by term gestational age group.**

|  | Participants and non-participants by term gestational age group (n=6172) | | | | | | | | | | | | | | | |
| --- | --- | --- | --- | --- | --- | --- | --- | --- | --- | --- | --- | --- | --- | --- | --- | --- |
|  | Early term (37-38 weeks) | | | | Full term (39-40 weeks) | | | | Late term (41 weeks) | | | | Post term (42-43.6 weeks) | | | |
|  | Participants  (n=797) | | Non-participants  (n=475) | | Participants  (n=1976) | | Non-participants  (n=1200) | | Participants  (n=737) | | Non-participants  (n=466) | | Participants  (n=327) | | Non-participants  (n=194) | |
| Motor impairment (moderate/much)^a^, n (%) | 7 | (0.9) | * | * | 16 | (0.8) | 9 | (0.8) | * | * | * | * | 7 | (2.1) | * | * |
| Visual impairment (moderate/much)^b^, n (%) | 91 | (11.4) | 52 | (10.9) | 225 | (11.4) | 129 | (10.8) | 65 | (8.8) | 43 | (9.2) | 38 | (11.6) | 19 | (9.8) |
| Hearing impairment (moderate/much)^c^, n (%) | 11 | (1.4) | 9 | (1.9) | 41 | (2.1) | 31 | (2.6) | 15 | (2.0) | 13 | (2.8) | 7 | (2.1) | * | * |
| Outside school: Playing sports or exercise so that you get out of breath or sweat for ≥2 hours/week^d^, n (%) | 538 | (68.5) | 300 | (65.1) | 1358 | (69.9) | 763 | (65.3) | 506 | (69.9) | 295 | (65.6) | 235 | (72.3) | 125 | (65.4) |
| Partake in organized training once a week or more^e^, n (%) | 447 | (57.5) | 205 | (45.1) | 1117 | (57.6) | 531 | (45.9) | 405 | (55.9) | 204 | (45.6) | 169 | (52.2) | 86 | (44.6) |
| Partake in non-organized training with others once a week or more^f^, n (%) | 403 | (51.9) | 216 | (47.6) | 971 | (50.1) | 592 | (50.9) | 373 | (51.8) | 234 | (52.1) | 163 | (50.8) | 89 | (46.4) |
| Exercise on your own once a week or more^g^, n (%) | 493 | (63.1) | 284 | (61.7) | 1205 | (62.1) | 712 | (61.1) | 441 | (60.8) | 284 | (63.0) | 201 | (62.2) | 106 | (55.2) |

*Exact number not shown for counts below 5 due to privacy reasons.

Abbreviation: Young-HUNT4=Trøndelag Health Study 2017-2019 (13-19 years), Survey 4.

^a^Data missing for 24 early term participants, 18 early term non-participants, 60 full term participants, 56 full term non-participants, 28 late term participants, 19 late term non-participants, eight post term participants and nine post term non-participants.

^b^Data missing for twelve early term participants, ten early term non-participants, 25 full term participants, 27 full term non-participants, 9 late term participants, 7 late term non-participants, one post term participant and seven post term non-participants.

^c^Data missing for 28 early term participants, 17 early term non-participants, 51 full term participants, 51 full term non-participants, 30 late term participants, 18 late term non-participants, 6 post term participants and 9 post term non-participants.

^d^Data missing for twelve early term participants, 14 early term non-participants, 34 full term participants, 31 full term non-participants, 13 late term participants, 16 late term non-participants, two post term participants and three post term non-participants.

^e^Data missing for 19 early term participants, 20 early term non-participants, 38 full term participants, 42 full term non-participants, 12 late term participants, 19 late term non-participants, three post term participants and one post term non-participant.

^f^Data missing for 21 early term participants, 21 early term non-participants, 37 full term participants, 38 full term non-participants, 17 late term participants, 17 late term non-participants, six post term participants and two post term non-participants.

^g^Data missing for 16 early term participants, 15 early term non-participants, 35 full term participants, 34 full term non-participants, twelve late term participants, 15 late term non-participants, four post term participants and two post term non-participants.
