# Supplementary material for: Gestational age and device-measured physical activity in adolescents and adults: A linkage of the HUNT Study and Medical Birth Registry
Source: PLoS One. 2026 Sep 23;21(9):e0357277. doi: 10.1371/journal.pone.0357277 (PMC13600527; doi:10.1371/journal.pone.0357277)
Supplement: Supplementary file 6 [file pone.0357277.s008.docx]

**S6 Table.** **Background data of study participants and non-participants in HUNT4 by preterm gestational age group.**

|  | Participants and non-participants by preterm gestational age groups (n=853) | | | | | | | | | | | |
| --- | --- | --- | --- | --- | --- | --- | --- | --- | --- | --- | --- | --- |
|  | Very preterm (<32 weeks) | | | | Moderately preterm (32-33 weeks) | | | | Late preterm (34-36 weeks) | | | |
|  | Participants  (n=37) | | Non-participants  (n=42) | | Participants  (n=45) | | Non-participants  (n=53) | | Participants  (n=272) | | Non-participants  (n=404) | |
| Education^a^, n (%) |  |  |  |  |  |  |  |  |  |  |  |  |
| Lower secondary | * | * | * | * | * | * | * | * | 5 | (1.8) | 21 | (5.2) |
| Upper secondary | * | * | * | * | * | * | * | * | 12 | (4.4) | 37 | (9.2) |
| Upper secondary, extended | * | * | 8 | (19.0) | 7 | (15.6) | 9 | (17.3) | 42 | (15.4) | 57 | (14.1) |
| Short-cycle tertiary | 6 | (16.2) | 13 | (31.0) | 12 | (26.7) | 17 | (32.7) | 70 | (25.7) | 112 | (27.8) |
| Lower tertiary (Bachelor) | 15 | (40.5) | 8 | (19.0) | 14 | (31.1) | 18 | (34.6) | 75 | (27.6) | 96 | (23.8) |
| Upper tertiary (Master) | 7 | (18.9) | 9 | (21.4) | 8 | (17.8) | * | * | 68 | (25.0) | 80 | (19.9) |
| Long-term illness/injury of a physical or psychological nature^b^, n (%) | 15 | (40.5) | 15 | (35.7) | 18 | (40.0) | 17 | (32.1) | 78 | (28.7) | 136 | (33.7) |
| Exercise for once a week or more^c^, n (%) | 32 | (88.9) | 33 | (80.5) | 38 | (86.4) | 36 | (70.6) | 231 | (86.5) | 317 | (79.4) |
| Moderate or vigorous intensity when exercising (if exercising once a week or more)^d^, n (%) | 25 | (78.1) | 22 | (66.7) | 32 | (84.2) | 30 | (83.3) | 173 | (75.5) | 246 | (78.1) |
| Duration of 30 minutes or more when exercising (if exercising once a week or more)?^e^, n (%) | 28 | (87.5) | 25 | (78.1) | 29 | (76.3) | 33 | (91.7) | 193 | (83.9) | 253 | (80.1) |

*Exact number not shown for counts below 5 due to privacy reasons.

Abbreviation: HUNT4=Trøndelag Health Study 2017-2019, Survey 4.

^a^Data missing for one moderately preterm non-participant and one late preterm non-participant.

^b^Data missing for one late preterm participant and two late preterm non-participants.

^c^Data missing for one very preterm participant, one very preterm non-participant, one moderately preterm participant, two moderately preterm non-participants, five late preterm participants and five late preterm non-participants.

^d^Data missing for five very preterm participants, nine very preterm non-participants, seven moderately preterm participants, 17 moderately preterm non-participants, 43 late preterm participants and 89 late preterm non-participants.

^e^Data missing for five very preterm participants, ten very preterm non-participants, seven moderately preterm participants, 17 moderately preterm non-participants, 42 late preterm participants and 88 late preterm non-participants.
