# Supplementary material for: Gestational age and device-measured physical activity in adolescents and adults: A linkage of the HUNT Study and Medical Birth Registry
Source: PLoS One. 2026 Sep 23;21(9):e0357277. doi: 10.1371/journal.pone.0357277 (PMC13600527; doi:10.1371/journal.pone.0357277)
Supplement: Supplementary file 7 [file pone.0357277.s009.docx]

**S7 Table.** **Background data of study participants and non-participants in HUNT4 by term gestational age group.**

|  | Participants and non-participants by term gestational age group (n=17 848) | | | | | | | | | | | | | | | |
| --- | --- | --- | --- | --- | --- | --- | --- | --- | --- | --- | --- | --- | --- | --- | --- | --- |
|  | Early term (37-38 weeks) | | | | Full term (39-40 weeks) | | | | Late term (41 weeks) | | | | Post term (42-43.6 weeks) | | | |
|  | Participants  (n=1001) | | Non-participants  (n=1218) | | Participants  (n=4144) | | Non-participants  (n=4623) | | Participants  (n=2008) | | Non-participants  (n=2239) | | Participants  (n=1285) | | Non-participants  (n=1330) | |
| Education^a^, n (%) |  |  |  |  |  |  |  |  |  |  |  |  |  |  |  |  |
| Lower secondary | 22 | (2.2) | 41 | (3.4) | 70 | (1.7) | 134 | (2.9) | 42 | (2.1) | 55 | (2.5) | 23 | (1.8) | 42 | (3.2) |
| Upper secondary | 48 | (4.8) | 116 | (9.6) | 272 | (6.6) | 396 | (8.6) | 109 | (5.4) | 211 | (9.5) | 80 | (6.2) | 110 | (8.3) |
| Upper secondary, extended | 164 | (16.4) | 242 | (20.0) | 610 | (14.8) | 807 | (17.6) | 345 | (17.2) | 396 | (17.8) | 184 | (14.4) | 243 | (18.3) |
| Short-cycle tertiary | 233 | (23.3) | 293 | (24.3) | 930 | (22.5) | 1139 | (24.8) | 424 | (21.2) | 538 | (24.1) | 314 | (24.5) | 307 | (23.2) |
| Lower tertiary (Bachelor) | 264 | (26.4) | 282 | (23.3) | 1118 | (27.0) | 1106 | (24.1) | 517 | (25.8) | 546 | (24.5) | 357 | (27.8) | 305 | (23.0) |
| Upper tertiary (Master) | 269 | (26.9) | 234 | (19.4) | 1134 | (27.4) | 1008 | (22.0) | 564 | (28.2) | 482 | (21.6) | 324 | (25.3) | 319 | (24.1) |
| Long-term illness/injury of a physical or psychological nature^b^, n (%) | 269 | (26.9) | 371 | (30.5) | 1146 | (27.7) | 1413 | (30.6) | 549 | (27.3) | 675 | (30.1) | 367 | (28.6) | 452 | (34.0) |
| Exercise for once a week or more^c^, n (%) | 811 | (81.4) | 971 | (81.0) | 3419 | (83.3) | 3670 | (80.3) | 1659 | (83.4) | 1777 | (80.7) | 1078 | (84.7) | 1048 | (79.3) |
| Moderate or vigorous intensity when exercising (if exercising once a week or more)^d^, n (%) | 617 | (76.6) | 732 | (75.7) | 2702 | (79.3) | 2807 | (76.8) | 1269 | (76.9) | 1386 | (78.4) | 872 | (81.4) | 806 | (77.2) |
| Duration of 30 minutes or more when exercising (if exercising once a week or more)?^e^, n (%) | 687 | (84.9) | 794 | (82.0) | 2915 | (85.4) | 3063 | (83.7) | 1393 | (84.3) | 1449 | (81.8) | 933 | (86.6) | 854 | (81.6) |

Abbreviation: HUNT4=Trøndelag Health Study 2017-2019, Survey 4.

^a^Data missing for one early term participant, ten early term non-participants, ten full term participants, 33 full term non-participants, seven late term participants, 11 late term non-participants, three post term participants and four post term non-participants.

^b^Data missing for eight early term participants, ten early term non-participants, 31 full term participants, 26 full term non-participants, 16 late term participants, 20 late term non-participants, eight post term participants and six post term non-participants.

^c^Data missing for five early term participants, 19 early term non-participants, 41 full term participants, 54 full term non-participants, 18 late term participants, 36 late term non-participants, 13 post term participants and 8 post term non-participants.

^d^Data missing for 195 early term participants, 251 early term non-participants, 738 full term participants, 970 full term non-participants, 357 late term participants, 471 late term non-participants, 214 post term participants and 286 post term non-participants.

^e^Data missing for 192 early term participants, 250 early term non-participants, 732 full term participants, 963 full term non-participants, 355 late term participants, 468 late term non-participants and 208 post term participants and 284 post term non-participants.
