# Supplementary material for: Gestational age and device-measured physical activity in adolescents and adults: A linkage of the HUNT Study and Medical Birth Registry
Source: PLoS One. 2026 Sep 23;21(9):e0357277. doi: 10.1371/journal.pone.0357277 (PMC13600527; doi:10.1371/journal.pone.0357277)
Supplement: Supplementary file 8 [file pone.0357277.s010.docx]

**S8 Table.** **Linear association of metabolic equivalent of task (MET) min/day of physical activity categories by 100 grams increase in birth weight among adolescents and adults.**

| **Total sample** | n=12 896 | | | Females  n=7752 | | | Males  n=5144 | | |
| --- | --- | --- | --- | --- | --- | --- | --- | --- | --- |
|  | B | (95% CI)^a^ | p-value | B | (95% CI)^b^ | p-value | B | (95% CI)^b^ | p-value |
| Total PA | -0.29 | (-0.88 to 0.30) | 0.339 | -0.30 | (-1.08 to 0.50) | 0.430 | -0.28 | (-1.19 to 0.63) | 0.569 |
| MVPA | -0.08 | (-0.35 to 0.21) | 0.570 | -0.26 | (-0.59 to 0.08) | 0.128 | 0.18 | (-0.23 to 0.58) | 0.408 |
| Light PA | -0.22 | (-0.67 to 0.24) | 0.373 | -0.04 | (-0.67 to 0.63) | 0.915 | -0.47 | (-1.23 to 0.33) | 0.237 |
| **Young-HUNT4** | n=4104 | | | Females  n=2469 | | | Males  n=1635 | | |
|  | B | (95% CI)^a^ | p-value | B | (95% CI)^b^ | p-value | B | (95% CI)^b^ | p-value |
| Total PA | -0.004 | (-0.80 to 0.75) | 0.988 | 0.06 | (-0.97 to 1.00) | 0.896 | -0.09 | (-1.36 to 1.21) | 0.890 |
| MVPA | -0.16 | (-0.60 to 0.26) | 0.478 | -0.40 | (-0.93 to 0.10) | 0.147 | 0.15 | (-0.56 to 0.86) | 0.681 |
| Light PA | 0.16 | (-0.36 to 0.66) | 0.610 | 0.47 | (-0.25 to 1.12) | 0.200 | -0.24 | (-1.14 to 0.67) | 0.600 |
| **HUNT4** | n=8792 | | | Females  n=5283 | | | Males  n=3509 | | |
|  | B | (95% CI)^a^ | p-value | B | (95% CI)^b^ | p-value | B | (95% CI)^b^ | p-value |
| Total PA | -0.39 | (-1.19 to 0.42) | 0.321 | -0.43 | (-1.34 to 0.50) | 0.375 | -0.35 | (-1.60 to 0.92) | 0.592 |
| MVPA | 0.04 | (-0.29 to 0.37) | 0.804 | -0.13 | (-0.54 to 0.26) | 0.513 | 0.27 | (-0.28 to 0.86) | 0.314 |
| Light PA | -0.43 | (-1.11 to 0.24) | 0.189 | -0.30 | (-1.10 to 0.49) | 0.456 | -0.62 | (-1.66 to 0.35) | 0.238 |

Abbreviations: CI=confidence interval; HUNT4=Trøndelag Health Study 2017-2019, Survey 4; MET=metabolic equivalent of task; MVPA=moderate to vigorous physical activity; PA=physical activity; Young-HUNT4=Trøndelag Health Study 2017-2019 (13-19 years), Survey 4.

^a^Based on bootstrapped regression analysis with birth weight as independent variable, adjusted for age and sex.

^b^Based on bootstrapped regression analysis with birth weight as independent variable, adjusted for age.
