# Supplementary material for: Gestational age and device-measured physical activity in adolescents and adults: A linkage of the HUNT Study and Medical Birth Registry
Source: PLoS One. 2026 Sep 23;21(9):e0357277. doi: 10.1371/journal.pone.0357277 (PMC13600527; doi:10.1371/journal.pone.0357277)
Supplement: Supplementary file 9 [file pone.0357277.s011.docx]

**S9 Table.** **Linear association of min/day of physical activity categories by 100 grams increase in birth weight among adolescents and adults.**

| **Total sample** | n=12 896 | | | Females  n=7752 | | | Males  n=5144 | | |
| --- | --- | --- | --- | --- | --- | --- | --- | --- | --- |
|  | B | (95% CI)^a^ | p-value | B | (95% CI)^b^ | p-value | B | (95% CI)^b^ | p-value |
| Total PA | -0.12 | (-0.40 to 0.17) | 0.407 | -0.05 | (-0.44 to 0.36) | 0.808 | -0.23 | (-0.68 to 0.24) | 0.337 |
| MVPA | -0.008 | (-0.07 to 0.06) | 0.803 | -0.05 | (-0.13 to 0.03) | 0.186 | 0.05 | (-0.04 to 0.14) | 0.314 |
| Light PA | -0.11 | (-0.37 to 0.15) | 0.411 | 0.004 | (-0.36 to 0.39) | 0.982 | -0.28 | (-0.71 to 0.16) | 0.206 |
| **Young-HUNT4** | n=4104 | | | Females  n=2469 | | | Males  n=1635 | | |
|  | B | (95% CI)^a^ | p-value | B | (95% CI)^b^ | p-value | B | (95% CI)^b^ | p-value |
| Total PA | 0.03 | (-0.32 to 0.36) | 0.871 | 0.17 | (-0.28 to 0.60) | 0.475 | -0.15 | (-0.73 to 0.44) | 0.631 |
| MVPA | -0.02 | (-0.12 to 0.07) | 0.652 | -0.07 | (-0.19 to 0.04) | 0.251 | 0.04 | (-0.12 to 0.21) | 0.617 |
| Light PA | 0.06 | (-0.25 to 0.35) | 0.760 | 0.24 | (-0.16 to 0.63) | 0.251 | -0.19 | (-0.70 to 0.34) | 0.482 |
| **HUNT4** | n=8792 | | | Females  n=5283 | | | Males  n=3509 | | |
|  | B | (95% CI)^a^ | p-value | B | (95% CI)^b^ | p-value | B | (95% CI)^b^ | p-value |
| Total PA | -0.20 | (-0.58 to 0.19) | 0.297 | -0.15 | (-0.61 to 0.31) | 0.547 | -0.28 | (-0.89 to 0.33) | 0.378 |
| MVPA | 0.02 | (-0.06 to 0.09) | 0.700 | -0.03 | (-0.12 to 0.07) | 0.560 | 0.07 | (-0.06 to 0.21) | 0.259 |
| Light PA | -0.22 | (-0.58 to 0.17) | 0.232 | -0.12 | (-0.57 to 0.32) | 0.605 | -0.35 | (-0.93 to 0.19) | 0.231 |

Abbreviations: CI=confidence interval; HUNT4=Trøndelag Health Study 2017-2019, Survey 4; MVPA=moderate to vigorous physical activity; PA=physical activity; Young-HUNT4=Trøndelag Health Study 2017-2019 (13-19 years), Survey 4.

^a^Based on bootstrapped regression analysis with birth weight as independent variable, adjusted for age and sex.

^b^Based on bootstrapped regression analysis with birth weight as independent variable, adjusted for age.
