# Supplementary material for: Gestational age and device-measured physical activity in adolescents and adults: A linkage of the HUNT Study and Medical Birth Registry
Source: PLoS One. 2026 Sep 23;21(9):e0357277. doi: 10.1371/journal.pone.0357277 (PMC13600527; doi:10.1371/journal.pone.0357277)
Supplement: Supplementary file 10 [file pone.0357277.s012.docx]

**S10 Table.** **Linear association of metabolic equivalent of task (MET) min/day of physical activity categories by increase in gestational age in weeks among** **adolescents and adults with additional adjustment for body mass index.**

| **Total sample** | n=12 855* | | | Females  n=7724* | | | Males  n=5131* | | |
| --- | --- | --- | --- | --- | --- | --- | --- | --- | --- |
|  | B | (95% CI)^a^ | p-value | B | (95% CI)^b^ | p-value | B | (95% CI)^b^ | p-value |
| Total PA | **1.80** | **(0.03 to 3.61)** | 0.040 | 1.85 | (-0.25 to 4.11) | 0.097 | 1.84 | (-0.81 to 4.50) | 0.209 |
| MVPA | 0.14 | (-0.65 to 0.91) | 0.723 | -0.48 | (-1.52 to 0.55) | 0.352 | 0.97 | (-0.25 to 2.18) | 0.129 |
| Light PA | **1.67** | **(0.23 to 3.13)** | 0.020 | **2.33** | **(0.59 to 4.10)** | 0.011 | 0.87 | (-1.30 to 2.92) | 0.481 |
| **Young-HUNT4** | n=4077* | | | Females  n=2451* | | | Males  n=1626* | | |
|  | B | (95% CI)^a^ | p-value | B | (95% CI)^b^ | p-value | B | (95% CI)^b^ | p-value |
| Total PA | **2.56** | **(0.01 to 5.11)** | 0.044 | 2.24 | (-0.70 to 5.00) | 0.147 | 3.08 | (-0.99 to 7.45) | 0.169 |
| MVPA | 0.89 | (-0.44 to 2.26) | 0.177 | 0.38 | (-1.21 to 1.86) | 0.620 | 1.55 | (-0.51 to 3.65) | 0.157 |
| Light PA | 1.67 | (-0.01 to 3.45) | 0.055 | 1.86 | (-0.21 to 3.80) | 0.077 | 1.53 | (-1.30 to 4.50) | 0.328 |
| **HUNT4** | n=8778* | | | Females  n=5273* | | | Males  n=3505* | | |
|  | B | (95% CI)^a^ | p-value | B | (95% CI)^b^ | p-value | B | (95% CI)^b^ | p-value |
| Total PA | 0.36 | (-1.95 to 2.52) | 0.748 | 0.24 | (-2.48 to 2.90) | 0.847 | 0.49 | (-3.27 to 4.27) | 0.797 |
| MVPA | 0.38 | (-0.67 to 1.38) | 0.464 | -0.15 | (-1.33 to 1.14) | 0.827 | 1.10 | (-0.38 to 2.54) | 0.130 |
| Light PA | -0.02 | (-1.92 to 1.81) | 0.986 | 0.39 | (-1.85 to 2.54) | 0.753 | -0.62 | (-3.69 to 2.50) | 0.707 |

*Participants with available data on age, sex and body mass index (kg/m^2^).

Abbreviations: CI=confidence interval; HUNT4=Trøndelag Health Study 2017-2019, Survey 4; MET=metabolic equivalent of task; MVPA=moderate to vigorous physical activity; PA=physical activity; Young-HUNT4=Trøndelag Health Study 2017-2019 (13-19 years), Survey 4.

^a^Based on bootstrapped regression analysis with gestational age as independent variable, adjusted for age, sex and body mass index.

^b^Based on bootstrapped regression analysis with gestational age as independent variable, adjusted for age and body mass index.

Estimates with 95% confidence intervals not including zero are presented in bold.
