# Supplementary material for: Gestational age and device-measured physical activity in adolescents and adults: A linkage of the HUNT Study and Medical Birth Registry
Source: PLoS One. 2026 Sep 23;21(9):e0357277. doi: 10.1371/journal.pone.0357277 (PMC13600527; doi:10.1371/journal.pone.0357277)
Supplement: Supplementary file 11 [file pone.0357277.s013.docx]

**S11 Table. Linear association of min/day of physical activity categories by increase in gestational age in weeks among adolescents and adults with additional adjustment for body mass index.**

| **Total sample** | n=12 855* | | | Females  n=7724* | | | Males  n=5131* | | |
| --- | --- | --- | --- | --- | --- | --- | --- | --- | --- |
|  | B | (95% CI)^a^ | p-value | B | (95% CI)^b^ | p-value | B | (95% CI)^b^ | p-value |
| Total PA | **1.08** | **(0.21 to 1.96)** | 0.014 | **1.42** | **(0.38 to 2.51)** | 0.009 | 0.71 | (-0.59 to 2.01) | 0.327 |
| MVPA | 0.02 | (-0.17 to 0.19) | 0.854 | -0.11 | (-0.35 to 0.13) | 0.360 | 0.19 | (-0.10 to 0.46) | 0.220 |
| Light PA | **1.07** | **(0.27 to 1.88)** | 0.011 | **1.53** | **(0.56 to 2.53)** | 0.002 | 0.52 | (-0.69 to 1.69) | 0.449 |
| **Young-HUNT4** | n=4077* | | | Females  n=2451* | | | Males  n=1626* | | |
|  | B | (95% CI)^a^ | p-value | B | (95% CI)^b^ | p-value | B | (95% CI)^b^ | p-value |
| Total PA | **1.10** | **(0.03 to 2.22)** | 0.054 | 1.18 | (-0.13 to 2.44) | 0.087 | 1.04 | (-0.81 to 3.05) | 0.298 |
| MVPA | 0.20 | (-0.11 to 0.51) | 0.178 | 0.10 | (-0.26 to 0.44) | 0.576 | 0.33 | (-0.14 to 0.81) | 0.189 |
| Light PA | 0.90 | (-0.04 to 1.87) | 0.072 | 1.09 | (-0.12 to 2.20) | 0.074 | 0.71 | (-0.91 to 2.48) | 0.414 |
| **HUNT4** | n=8778* | | | Females  n=5273* | | | Males  n=3505* | | |
|  | B | (95% CI)^a^ | p-value | B | (95% CI)^b^ | p-value | B | (95% CI)^b^ | p-value |
| Total PA | 0.22 | (-0.92 to 1.31) | 0.689 | 0.41 | (-0.94 to 1.77) | 0.564 | -0.06 | (-1.84 to 1.81) | 0.955 |
| MVPA | 0.06 | (-0.17 to 0.30) | 0.577 | -0.04 | (-0.31 to 0.25) | 0.785 | 0.20 | (-0.15 to 0.55) | 0.232 |
| Light PA | 0.16 | (-0.88 to 1.18) | 0.759 | 0.45 | (-0.85 to 1.74) | 0.515 | -0.26 | (-1.94 to 1.51) | 0.770 |

*Participants with available data on age, sex and body mass index (kg/m^2^).

Abbreviations: CI=confidence interval; HUNT4=Trøndelag Health Study 2017-2019, Survey 4; MVPA=moderate to vigorous physical activity; PA=physical activity; Young-HUNT4=Trøndelag Health Study 2017-2019 (13-19 years), Survey 4.

^a^Based on bootstrapped regression analysis with gestational age as independent variable, adjusted for age, sex and body mass index.

^b^Based on bootstrapped regression analysis with gestational age as independent variable, adjusted for age and body mass index.

Estimates with 95% confidence intervals not including zero are presented in bold.
